# Neurovascular coupling over cortical brain areas and resting state network connectivity with and without rigidified carotid artery

**DOI:** 10.1117/1.NPh.12.S1.S14606

**Published:** 2025-02-04

**Authors:** Marleen E. Bakker, Cong Zhang, Matthieu P. Vanni, Frédéric Lesage

**Affiliations:** a Institute of Biomedical Engineering, École Polytechnique de Montréal, Montreal, Quebec, Canada; b Université de Montréal, École d’Optométrie, Montreal, Quebec, Canada; c Institut Cardiologie de Montréal, Montreal, Quebec, Canada

**Keywords:** GCaMP, intrinsic optical imaging, neurovascular coupling, resting state

## Abstract

**Significance:**

Neurovascular coupling (NVC) is key to research as hemodynamics can reflect neuronal activation and is often used in studies regarding the resting state network (RSN). However, several circumstances, including diseases that reduce blood vessel elasticity, can diminish NVC. In these cases, hemodynamic proxies might not accurately reflect the neuronal RSN.

**Aim:**

We aim to investigate in resting state if (1) NVC differs over brain regions, (2) NVC remains intact with a mild rigidification of the carotid artery, (3) hemodynamic-based RSN reflects neuronal-based RSN, and (4) RSN differs with a mildly rigidified artery.

**Approach:**

We rigidified the right common carotid artery of mice (n=15) by applying a ${\mathrm{CaCl}}_{2}$-soaked cloth to it (NaCl for Sham, n=17). With simultaneous GCaMP and intrinsic optical imaging, we compared neuronal activation to hemodynamic changes over the entire cortex.

**Results:**

NVC parameters did not differ between the CaCl and Sham groups. Likewise, GCaMP and hemodynamic RSN showed similar connections in both groups. However, the parameters of NVC differed over brain regions. Retrosplenial regions had a slower response and a higher HbR peak than sensory and visual regions, and the motor cortex showed less HbO influx than sensory and visual regions.

**Conclusions:**

NVC in a resting state differs over brain regions but is not altered by mild rigidification of the carotid artery.

## 1 Introduction

Activations in specific parts of the brain can be evoked with stimuli. However, even in the absence of stimuli, the brain follows a structured pattern of activity, forming a resting state network (RSN).^1^^–^^5^ These networks are relatively similar between subjects,^6^ with a high correlation between homotopic brain areas, and a lower correlation between brain regions underlying different functions, for example, areas involved in visual and motor processing.^3^

Due to the difficulty in directly measuring neuronal activation over the entire human brain, RSN studies in humans have been largely based on hemodynamic proxies of activation, such as measured with BOLD-fMRI. This method is based on the notion that there is an increase in cerebral blood flow and an influx in oxygenated hemoglobin (HbO) accompanying neuronal activity, referred to as neurovascular coupling (NVC). It has previously been shown that neurovascular coupling might differ between brain areas,^7^^,^^8^ which could be related to a difference in blood vessel structure.^8^ Although NVC is usually investigated as the evoked response to a stimulus, it has also been found in the resting state, where spontaneous activations of neurons in awake mice were followed by an influx of HbO, with a similar response and timing to evoked neuronal activity.^9^^–^^12^ To our knowledge, no study toward NVC differences in resting state over cortical brain structures has been done so far.

In healthy conditions, NVC has been proven to be reliable and consistent across subjects. However, NVC can be weakened or altered in different situations, for example, primary brain gliomas,^13^ diabetes,^14^^,^^15^ and pontine infarction.^16^ Not surprisingly, factors that affect the vasculature of the brain, such as hypertension^17^^–^^20^ and atherosclerosis,^21^ a disease that causes thickening and hardening of vessel walls,^22^ tend to decrease NVC as well. Furthermore, NVC is shown to be decreased in aging individuals, and NVC dysfunction is strongly associated with Alzheimer’s disease,^21^^,^^23^^,^^24^ and dementia,^21^^,^^25^ as well as cerebral small vessel disease,^26^ and is often suspected to be a main contributor to these diseases. One of the potential causes for NVC dysfunction is the decreased ability to attenuate the pulse wave velocity that is caused by the heartbeat.^27^ In healthy, young subjects, the aorta and carotid arteries are compliant with the pulse pressure associated with each heartbeat (the Windkessel effect), thereby dampening the pulsatile flow before reaching the smaller vessels. As a consequence of the stiffening of the main blood vessels, a larger pulse wave will reach the small vessels in the brain, which has been shown to damage their structural integrity by causing endothelial dysfunction and increased blood-brain barrier permeability.^27^^–^^29^

Many RSN studies are done with fMRI and thus rely on NVC to give accurate information about underlying neuronal processes. RSNs have been found to be altered in neurovascular diseases such as cerebral small vessel disease,^30^ concussion,^31^ and post-concussion syndrome,^32^ and as a result of carotid endarterectomy.^33^ Furthermore, RSNs differ in patients with mild cognitive impairment and Alzheimer’s disease,^34^^–^^36^ and this change might even be useful as a biomarker to distinguish between mild cognitive impairment and Alzheimer’s,^37^^,^^38^ or even a predictive factor for Alzheimer’s.^39^ However, as these measurements have been done with a proxy relying on NVC, it remains uncertain whether the alterations in RSN are due to a change in neuronal functional connectivity, or a neurovascular decoupling that causes oxygenated blood flow to misrepresent the neuronal activity.

### 1.1 Aims

The main aim of this paper is to investigate the effect of arterial stiffness on NVC and RSN connectivity. We use the mouse model as developed by Sadekova et al.,^40^ where the carotid artery of the mouse is rigidified. This model isolates the effect of rigidification of the artery from other factors that may combine and confound observations. Exploiting combined calcium and hemodynamic imaging, we investigated the parameters of NVC, such as timing and amount of HbO influx, over different brain regions during rest, and compared these findings with mice with a rigid carotid artery in awake conditions. Furthermore, we investigated the possible changes in RSN as a result of the rigidification of this artery.

## 2 Methods

### 2.1 Surgeries

#### 2.1.1 Animals

**Fig. 1:**
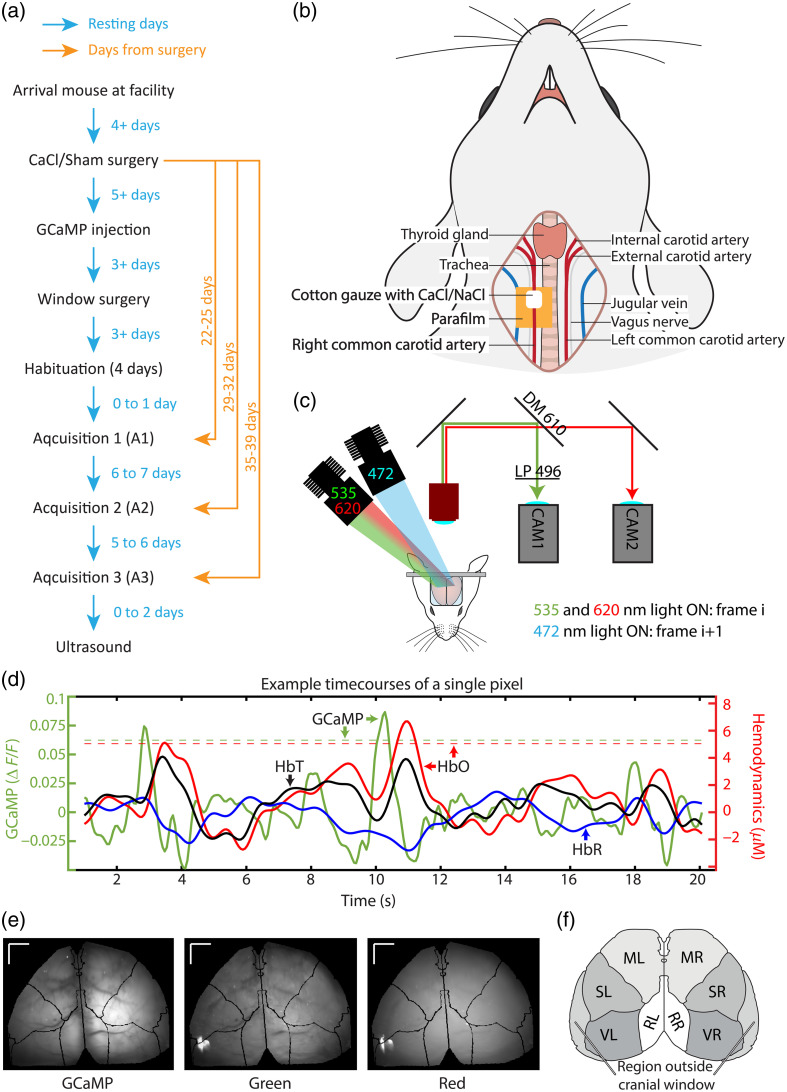
(a) Overview of the procedures that the mice were subjected to. (b) Schematic overview of the carotid artery calcification surgery. (c) Schematic overview of the imaging system. DM, dichroic mirror; LP, long pass filter. (d) Timecourses of a single pixel in the right sensory area of a single mouse. The GCaMP timecourse (in green) follows the y-axis on the left, whereas the hemodynamic timecourses (oxygenated hemoglobin—HbO—in red, deoxygenated hemoglobin—HbR—in blue, and total hemoglobin—HbT—in black) are on the right y-axis. The threshold for detecting spontaneous activation (z-score>1.95) for this pixel is depicted as a green dotted line for GCaMP, and a red dotted line for HbO. (e) Average images of the GCaMP, green, and red channels of a single acquisition. Black lines show the fitted Allen atlas. Lines in the top left corner depict the scale - lines are 1 mm. (f) Overview of the different regions of interest of the brain. VR, right visual area; SR, right sensory area; MR, right motor area; RR, right retrosplenial area; VL, left visual area; SL, left sensory area; ML, left motor area; RL, left retrosplenial area.

A total of 34 C57BL/6J mice were used in this study (16 male, 18 female), obtained from Charles River (Wilmington, Massachusetts, United States). After arrival in the animal facility, mice had at least 4 days of rest. Mice had ad libitum access to food and water and were housed under a 12-h light/dark schedule. All procedures were approved by the Animal Research Ethics Committee of the Montreal Heart Institute and conformed to the regulations of the Canadian Council on Animal Care. A general overview of the procedures undergone by one mouse can be seen in Fig. 1(a). In all mentions of anesthesia, mice were anesthetized with isoflurane (3% induction; 1.5% surgery in pure $O_{2}$), and their body temperature was controlled at 37°C with the help of an anal probe and a feedback-controlled heating pad. The heartrate, breathing, and body temperature were monitored continuously during this period (Small animal physiological monitoring system, Labeo Technologies Inc., Montréal, Canada).

#### 2.1.2 Calcium rigidification and GCaMP injection

The procedure followed here was the same as in Ref. 40. A piece of parafilm was immersed in glutaraldehyde for at least 10 h, after which it was rinsed with sterile saline. The mouse was anesthetized, and an incision of ∼1.5  cm was made at the midline in the neck to allow for isolation of the right common carotid artery. A sheet of parafilm was inserted below the artery, and a sterile cotton gauze soaked in 0.3M ${\mathrm{CaCl}}_{2}$ solution (or 0.9% NaCl for Sham) was held to the carotid artery for 20 min [Fig. 1(b)]. After the carotid treatment, the incision was closed with sutures and tissue adhesive (VetBond). Mice were treated with the local anesthetic Bupivacaine (Marcaine, 4  mg/kg SC infiltration at the incision site), the antibiotic Trimetoprim-Sulfa (Tribrissen; 30  mg/kg SC), and the anti-inflammatory Carprofen (Rimadyl; 5  mg/kg SC) immediately after surgery. Mice recovered for at least 2 days before GCaMP injection, for which mice were shortly anesthetized and injected with AAV2/php.eB-hSyn-GCaMP6s (CERVO molecular platform) in the tail vein with a catheter (50  μL, diluted with 150  μL of saline).

#### 2.1.3 Window surgery

Mice were anesthetized, ketoprofen (5  mg/kg, SC) was injected subcutaneously, and local analgesia (Lidocaine 0.2 mL of 4  mg/mL, SC) was administered on the top of the head. The head was shaved and cleaned with ethanol. The skin on top of the skull was cut away, and the tissue covering the skull was cleaned with a cotton swab. Vetbond was applied to the edges of the skin. A titanium headbar was fixed on the head with dental cement (C&B Metabond). The skull was covered with thin layers of cyanoacrylate glue. Carprofen (12.5  mg/kg, SC) was given right after the surgery, and Ketoprofen (5  mg/kg, SC) was administered the following day. The mouse rested for at least 3 days after the surgery.

#### 2.1.4 Imaging

Optical imaging was done on awake mice. To limit the stress that the mice could experience during imaging, they were habituated to head fixation and imaging over a period of 4 days, (15, 20, 30, and 45 min). Following habituation, intrinsic optical imaging (IOI) and GCaMP imaging were done simultaneously, at rest. IOI/GCaMP acquisitions lasted 10 min and were performed once per week for 3 weeks. These acquisitions will be referred to as A1, A2, and A3 and occurred on days 22 to 25, 29 to 32, and 35 to 39 after calcium rigidification surgery, respectively.

To excite the genetically encoded reporters, a blue light-emitting diode (LED) (472 nm) combined with a bandpass filter (FF02-472/30) was used to restrain the spectral excitation [Fig. 1(c)]. For the intrinsic optical imaging (IOI^41^), green (535 nm) and red (620 nm) LEDs were used intermittently with the blue LED. A two-camera design was chosen to simplify the dissociation of the three recorded signals. Camera 1 captures the green reflectance (∼535  nm) and the GCaMP emission (∼515  nm), whereas camera 2 captures the red reflectance (∼620  nm). To separate the red from the green/GCaMP, a dichroic (610 nm) was used. A long-pass filter (FF01-496/LP) was placed in front of camera 1 to filter out the fluorescence excitation of blue light. Two cameras were used (CS2100M, Thorlabs, Newton, New Jersey, United States), each with a framerate of 30 Hz. This yielded a final framerate of 15 Hz per color. Images were 512×512  pixels. Imaging was done with a LightTrack OiS200 system (Labeo Technologies Inc.). Analysis was done with the help of MATLAB (R2020b and R2023a). Code can be found on github.com/marl1bakker/CaCl_P2. The two cameras had a slight offset, which was corrected through an automatic coregistration of the first frame captured by each camera, followed by a manual verification. The system recorded images mirrored, and thus, a left–right flip was administered.

### 2.2 GCaMP and Hemodynamics

#### 2.2.1 Movement and outlier removal

Mice were fixed with their head bar during acquisition and placed on a treadmill, which registered locomotion (Fig. 7 in Appendix A). Several recordings were made, and the best session was chosen a posteriori with the help of the registered movements of the treadmill. Furthermore, during analysis, recordings of the movement of the treadmill were used to detect movement, and corresponding frames were marked, as were frames 1 s before and after the movement. These frames were excluded from the analysis. For a session to be included in the analysis, the threshold for the number of marked moved frames was set to 3000. All mice met this criterion for at least one session in all acquisitions. To detect frames with artifacts or movement that went undetected by the treadmill, an outlier detection was done on the processed data. All pixels over the brain were averaged per frame. Frames that diverted more than three standard deviations of the mean in either GCaMP or hemodynamic data were marked and excluded from the analysis. Last, an outlier detection of single pixels was done to remove pixels that were marked as an outlier on 1000 frames or more. This helped detect and exclude artifacts in the window, such as bubbles or scratches.

One mouse failed to show GCaMP expression and was thus excluded from all calculations that used GCaMP measurements. As the cranial window was of good quality and the hemodynamic measurements worked well for this mouse, it remained in the study for calculations that only included hemodynamic measurements. Two other mice showed window artifacts in acquisitions 2 and 3 that hindered the imaging of hemodynamic data. Because of this, the hemodynamic measurements for one mouse were excluded from acquisitions A2 and A3, and another mouse was only excluded from hemodynamic measurements of A3.

#### 2.2.2 ROI selection and image alignment

Regions of interest were determined by fitting the Allen atlas^42^ on top of the mouse brain, based on the location of bregma and lambda. Regions of the atlas were pooled into larger ROIs, resulting in left and right versions of motor (M), retrosplenial (R), sensory (S), visual (V), and auditory (A) regions [Figs. 1(e) and 1(f)]. Auditory regions were left out of the analysis because they often fell outside of the cranial window. To ensure the stability of the regions of interest over the distinct acquisitions over time, the Allen atlas was fitted on the first acquisition, and the second and third acquisitions were coregistered to the first. Coregistration was done using an affine 2D transformation where the mean squared error was used as an optimization parameter. Image registrations were checked manually. If automatic coregistration was off by more than 3 pixels, the coregistration was done by hand.

#### 2.2.3 Hemodynamic correction

The influx of blood that typically accompanies an increase in neuronal activation can cause a weakened recording of the GCaMP signal by absorbing both light that activates the GCaMP molecule and by absorbing the fluorescence that this molecule emits due to neuronal firing.^43^ Having information on hemodynamics allows for a correction for this alteration. Here, we used the same approach described by Valley et al.,^43^ in which a linear regression of the reflectance is used to remove the bulk of the interference.

#### 2.2.4 Normalization and filtering

A lowpass filter (cutting frequency at 3 Hz) is first applied to remove high-frequency noise. To remove slow drifts in the signal, we then normalized it by its own low-frequency content (lowpass filter with cutting frequency at 0.08 Hz), to get the fluorescence fluctuations (ΔF/F).

#### 2.2.5 Hemodynamic calculations

A lowpass filter of 1 Hz was applied to the green and red channels, after which it was normalized by a lowpass filter at 0.08 Hz to remove any non-functional variations from the signal. The light absorption of oxygenated hemoglobin (HbO) and deoxygenated hemoglobin (HbR) varies as a function of wavelength. The difference in reflectance of the green and red light thus allows us to calculate the changes in HbO and HbR in the same manner as Ref. 43, using the modified Beer–Lambert equation. Baseline values of HbO and HbR were set to 60 and 40  μM, respectively.^44^^–^^46^ Total hemoglobin levels (HbT) are calculated by adding HbO and HbR values.

#### 2.2.6 Neurovascular coupling

Spontaneous spikes in GCaMP activation, which were not provoked by any type of stimulation, were defined as fluorescence peaks that had a z-score of 1.95 or over [Fig. 1(d)]. Z-score was calculated as follows:

z=(x−μ)/σ,

where x is the GCaMP activation of a pixel, μ is the mean GCaMP activation of this pixel, and σ is the standard deviation of this pixel. For each spontaneous spike, the timecourses of HbO, HbR, and GCaMP were obtained from 5 s before to 10 s after the spike. All spontaneous spike timecourses of a single pixel were averaged. These time series were then used to investigate NVC over the whole brain by averaging the timecourses of all pixels over the brain and changes in NVC between regions of interest by averaging timecourses of all pixels within that region. Spontaneous spikes of HbO were calculated in a similar manner, where a z-score was calculated based on the HbO values on a pixel (x), the mean HbO activation of that pixel (μ), and the standard deviation of that pixel (σ). The threshold for a detected activation was likewise set to a z-score of 1.95 or over. The number of detected activations per brain area can be found in Figs. 8(a) and 8(b) in Appendix B.

#### 2.2.7 Ultrasound imaging

Ultrasound imaging (US) was done on a subset of mice (n=7 CaCl, n=6 Sham), using a transducer of 30 MHz. Mice were anesthetized and placed on a heating pad in a supine position. The hair on the neck was removed. Heartrate was measured with ECG. The right carotid artery was located with the help of the B-mode and the color Doppler.

Images were required in M-mode. The diameter of the vessel was calculated as the space between the brightly appearing vessel walls [Fig. 9(a) in Appendix C, for example frame]. Changes in vessel diameter were averaged over several (2 to 3) heartbeat cycles [green line in Fig. 9(a) in Appendix C]. The difference between the minimum and maximum diameters over this average curve was then used as a measurement. Anesthetized mice were sacrificed at the end of the ultrasound acquisition.

### 2.3 Statistics

#### 2.3.1 Movement

To verify that the CaCl and Sham groups did not differ in the amount of movement during acquisition, we compared the number of frames where movement was detected between the two groups. As the data were not normally distributed, a Kruskal–Wallis test was performed for each acquisition, none of which showed significance.

#### 2.3.2 NVC in Sham

To compare NVC between brain regions and groups, nine parameters were calculated and compared between regions of interest. First, the peak values of HbO, HbR, and GCaMP were compared [black triangles in Fig. 3(b), and top row of Fig. 3(c)]. Then, the increase in HbO and GCaMP was calculated as the difference between the peak and the preceding dip. For HbR, the decrease (difference between the peak and proceeding dip) was calculated [Fig. 3(b) and middle row of Fig. 3(c)]. Next, the time between the GCaMP peak and HbO peak was calculated and labeled “delay” [Fig. 3(b) and bottom left of Fig. 3(c)]. Last, the strength of the neurovascular coupling was calculated by dividing the HbO peak by the GCaMP peak [Fig. 3(c), bottom row, middle column] and by dividing the HbO increase by the GCaMP increase [Fig. 3(c), bottom row, right column].

Statistics were done on each parameter separately. First, an Anderson-darling test was done to determine if the data conformed to a normal distribution. If this was not the case, a non-parametric Friedman test was performed. Otherwise, an analysis of variance (ANOVA) was performed, and the sphericity of the assumptions was checked. If the sphericity assumption was not met, a Greenhouse–Geisser correction was performed. Because of the high number of statistical tests (especially relevant for statistics on the correlation matrices later in this article), there is a large chance of a false positive in the results. To correct this, a false discovery rate correction (FDR, Benjamini–Hochberg) was performed, providing us with adjusted p-values, hereafter referred to as q-values. Pairwise comparisons (Tukey–Kramer) were calculated on p-values that retained their significance after the FDR (q-values). Table 1 in Appendix D shows which tests were done for which parameter (A), and the p-values per seed pair of the post-hoc testing (B). Figure 10(a) in Appendix E shows NVC curves per mouse, per brain area.

#### 2.3.3 Ultrasound

The assumption of sampling from a normal distribution was checked with an Anderson–Darling test, and homogeneity of variance was tested with a two-sample F test. As both assumptions were met, CaCl and Sham groups were compared using a two-sample t-test [Fig. 9(b) in Appendix C].

#### 2.3.4 NVC with rigidified artery

The same parameters as NVC in Sham were calculated (Fig. 11 in Appendix F). A similar approach for determining correct tests as in NVC Sham was also used. However, the sample sizes between the groups differed (CaCl n=14, Sham n=17), and thus, a Mack–Skillings^47^ test (code from Pingel^48^) was used instead of a Friedman test in non-parametric instances. Which tests were used eventually can be found in Table 2 in Appendix G. None of the parameters showed a significant difference among groups, so no post-hoc tests were performed.

#### 2.3.5 Correlation Matrices

The correlation value of all seed pair combinations was calculated (Fig. 12 in Appendix H). The use of global signal regression (GSR) has been a topic of much debate in resting state studies.^49^ In this paper, the presented correlation matrices and statistics are without the use of GSR, to prevent the accidental removal of signal of interest. However, correlation matrices with GSR can be found in Fig. 13 in Appendix I. A Fisher’s z-transform was executed to calculate statistics.^50^ The data of the correlation matrices were not normally distributed (tested with the Kolmogorov–Smirnov test), and therefore, a non-parametric Mann–Whitney U test was performed for each ROI pair for comparisons between CaCl and Sham groups, and a Friedman test was chosen for tests over the three time points. An FDR was run on all seedpairs. To keep the possibility of seeing trends in the data, both p-values and q-values of the correlation matrixes can be found in Table 3 in Appendix J.

## 3 Results

### 3.1 Neurovascular Coupling in Sham—Spontaneous GCaMP Activation

**Fig. 2:**
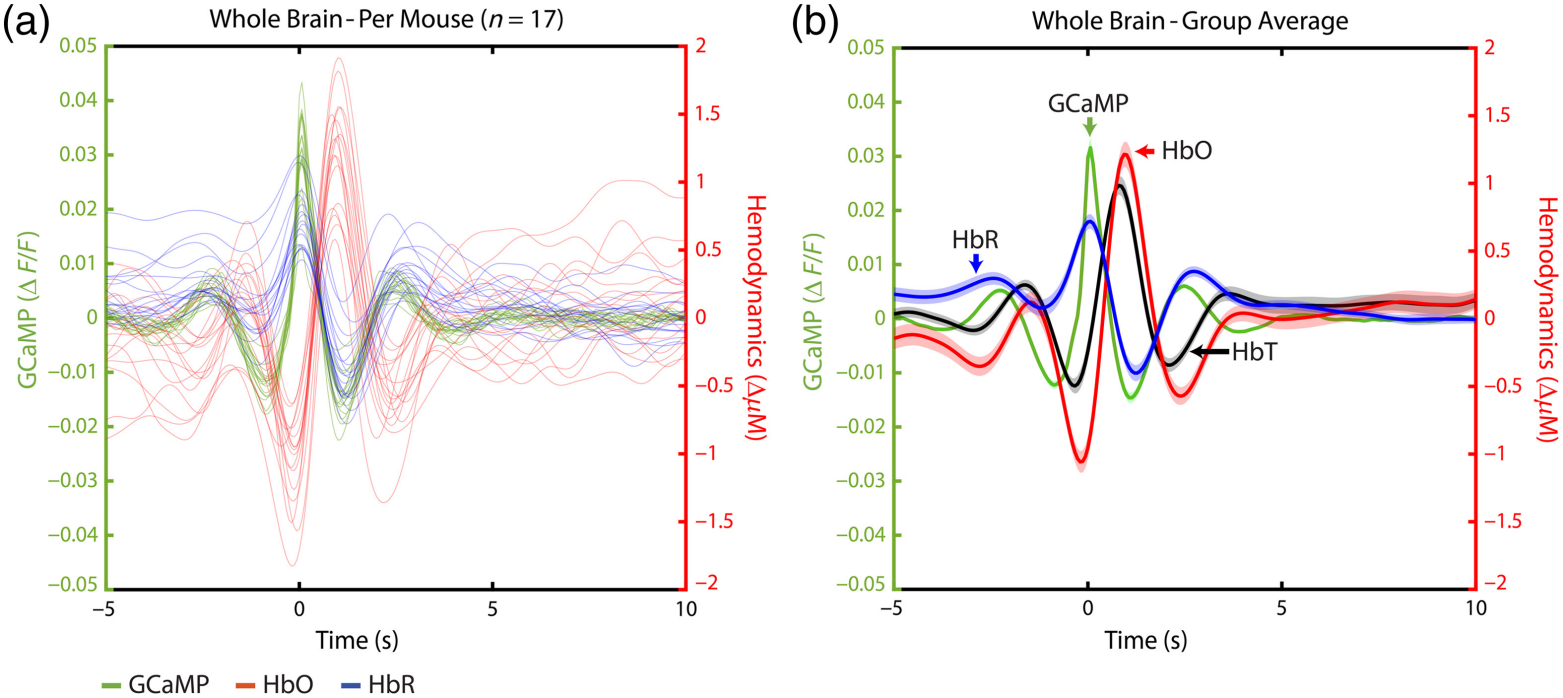
(a) Neurovascular coupling in individual mice. Each line depicts the average GCaMP, HbO, and HbR fluctuations 5 s before to 10 s after a detected spontaneous GCaMP activation. The green line depicts the GCaMP signal and corresponds to the left y-axis. Red lines depict HbO, and blue lines depict HbR, both corresponding to the right y-axis. Individual HbT curves are not shown to increase visibility. (b) Group average of the mice depicted in panel (a). The dark line shows the group average, and the lighter patches around the line show the standard error of the mean (SEM). See Figs. 10(b)–10(d) in Appendix E for plots with unfiltered data.

**Fig. 3:**
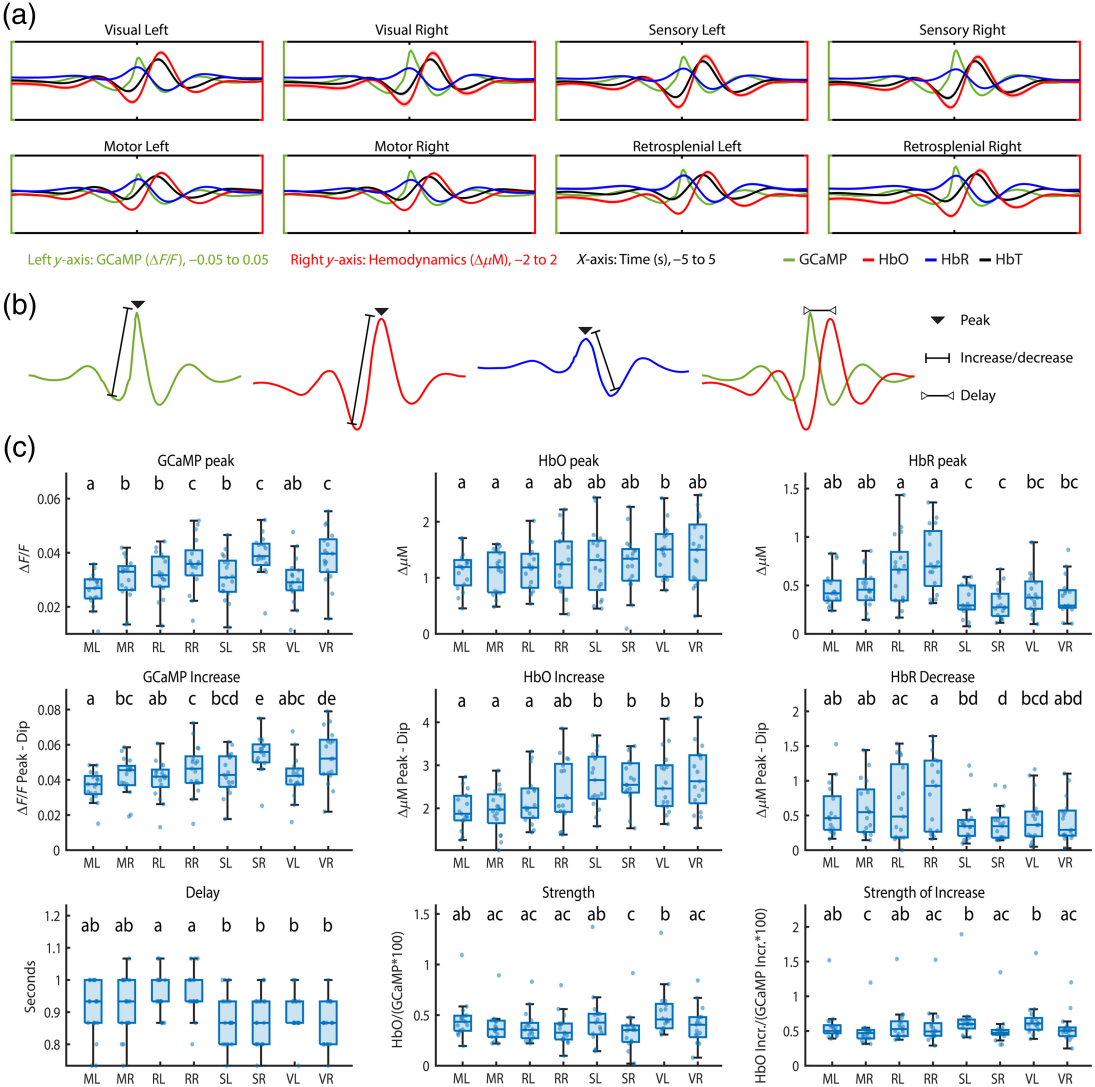
(a) Average curves for GCaMP (green), HbO (red), HbR (blue), and HbT (black) over different brain regions in the first acquisition of the Sham group. The left (green) y-axis depicts ΔF/F values for GCaMP, ranging from −0.05 to 0.05. The right (red) y-axis shows ΔμM for HbO, HbR, and HbT values, ranging from −2 to 2. The x-axis shows time, ranging from −5 to 5 s, with a notch at 0, where spontaneous GCaMP activation was detected. (b) Visual illustration of how the parameters in panel (c) were calculated. (c) Parameters related to NVC in different brain areas in the first acquisition of the Sham group. Letters above boxplots indicate significance: if two groups do not carry at least one similar letter, they differ significantly (p<0.05, see Table 1 in Appendix D).

The relation between neuronal activity and the hemodynamic response was investigated on the basis of spontaneous neuronal activity using GCaMP signal fluctuation as a proxy. These spontaneous events were detected, and the timecourses of the GCaMP, HbO, and HbR data from 5 s before to 10 s after each event were kept. The fluctuations were averaged over the whole brain (Fig. 2) and over separate brain areas (Fig. 3) for the mice in the Sham group (n=17) during the first acquisition.

As expected, we found neurovascular coupling to be present in the resting state, with a typical increase in HbO and a decrease in HbR after spontaneous GCaMP activity. As seen in Fig. 2(a) [and Fig. 10(a) in Appendix E], all mice show similar dynamics. The GCaMP signal tends to decrease before showing a quick peak. After the peak, the GCaMP shows an undershoot. This has been found before in cases without hemodynamic correction^51^^,^^52^ and could be caused by the increase in HbO hindering the GCaMP signal,^51^ but given that we did a correction for that in this study, this possibility is unlikely here. Last, the GCaMP signal fluctuates back to baseline. The GCaMP activity tends to stay much more stable around 0 in between activations, with less longer-term fluctuations, as can be seen in the hemodynamic measurements. The HbO and HbT timecourses follow a very similar pattern as GCaMP, delayed by ∼1  s, whereas the HbR timecourse shows an inverse pattern [Fig. 2(b)]. Both hemodynamic and GCaMP signals are back around baseline within 5 s. To exclude the possibility of the oscillations being caused by a filtering effect on the data, the same analysis was done on unfiltered data, showing very similar results [Figs. 10(b)–10(d) in Appendix E].

Next, in the same group of Sham mice (n=17), we investigated whether the NVC differed per brain area [Fig. 3(a)]. To do so, we calculated the average peaks of GCaMP, HbO, and HbR per mouse, per brain area [Fig. 3(c)]. However, as can be seen in Figs. 2(a) and 10(a) in Appendix E, the hemodynamic values tend to fluctuate and might start from a value that differed from 0. To focus on the change in hemodynamics in the moment of spontaneous GCaMP activation, without including longer-term hemodynamic fluctuations, we also calculated the increase in HbO, measured from the lowest to the highest point; the decrease in HbR, measured from the highest to the lowest point [Fig. 3(b)]; and the increase of HbO in relation to the increase in GCaMP. Statistical testing showed a significant difference between brain areas for all 9 parameters [Table 1(A) in Appendix D]. Post-hoc tests were conducted, and results can be seen in Fig. 3(c) [individual p-values in Table 1(B) in Appendix D].

First, when looking at the detected spontaneous activation of GCaMP and the increase of GCaMP, we see that, in all regions of interest, the right version showed a significantly higher peak than its left homotopic counterpart. The left motor region was significantly lower than all other left GCaMP peaks, and the same was true for the right motor region compared with the other right regions of interest. This significance disappeared when measured by the amount of increase.

The HbO peak and HbO increase show similar results, although the increase shows more significant differences than the peaks. In general, the sensory and visual areas show an increased HbO influx compared with the motor and retrosplenial areas, where all sensory and visual areas except for the right retrosplenial area differ significantly from all motor and retrosplenial areas when measured by the HbO increase.

The retrosplenial area shows the highest peak in HbR, as well as the largest decrease. In general, although not always significantly different, the same two groups as HbO can be distinguished, with the motor and retrosplenial regions showing a higher peak and larger decrease and the sensory and visual regions showing a lower peak and smaller decrease. Interestingly, neither HbO nor HbR results seem to show a difference between hemispheres, whereas this difference is very apparent in the GCaMP data.

The delay of the HbO peak compared with the GCaMP peak is the longest in the retrosplenial area and differed significantly from the shorter delays of the sensory and visual areas. The motor region did not differ significantly from any of the other regions.

The strength of the neurovascular coupling shows significant differences between the homotopic regions of the sensory and visual areas, both when measured at the peaks and when measured by increases. Furthermore, the homotopic regions of the motor cortex show a significant difference when measured by increase. These differences reflect the differences found in the GCaMP results.

### 3.2 Neurovascular Coupling in Sham—Spontaneous HbO Activation

**Fig. 4:**
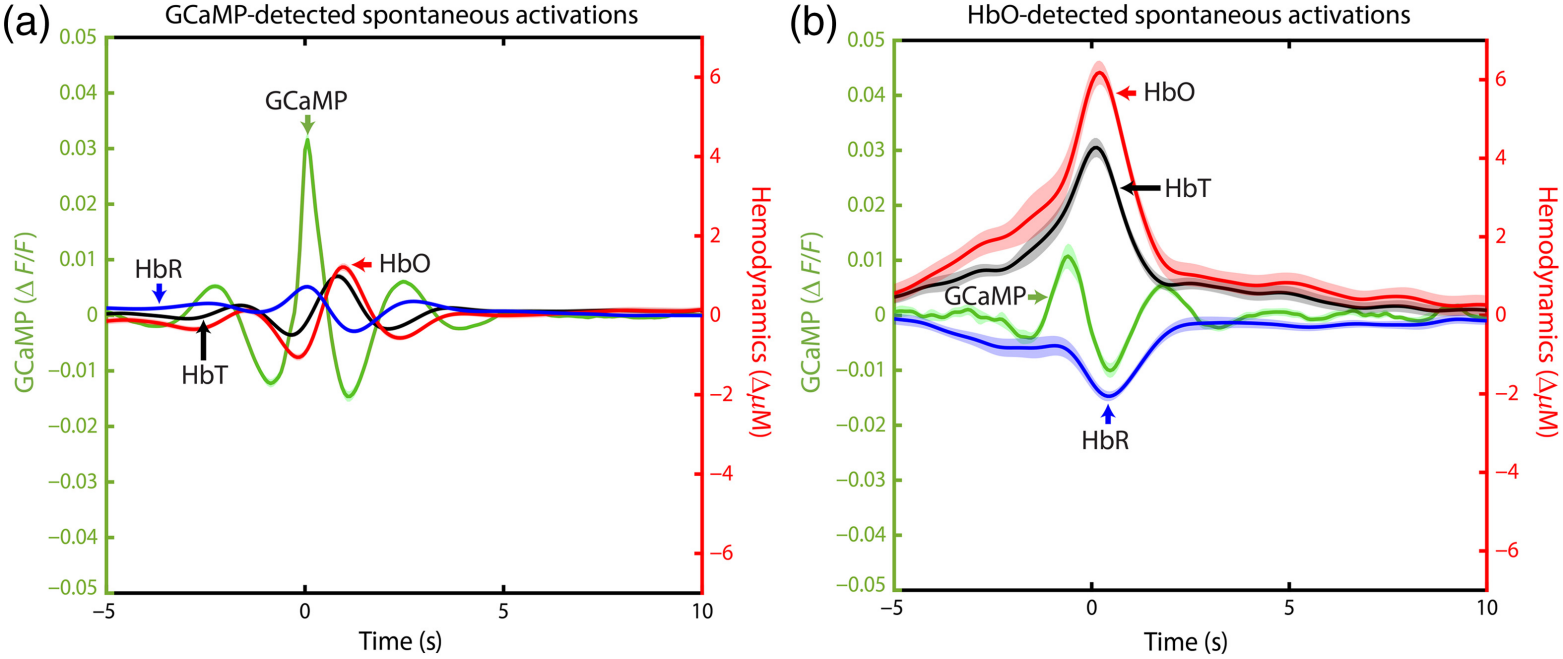
Neurovascular coupling for all Sham mice of Acquisition 1 over the whole brain. The green line depicts the GCaMP signal and corresponds to the left y-axis. Red lines depict HbO, blue lines depict HbR, and black lines depict HbT, all corresponding to the right y-axis. The dark line shows the group average, and the lighter patches around the line show the standard error of the mean (SEM). (a) Curves of detected spontaneous GCaMP activations, where 0 is the time of detection based on GCaMP data. Note that these data are the same as shown in Fig. 2(b), with adjusted y-axes to make a direct comparison to panel (b). (b) Curves of detected spontaneous HbO activations, where 0 is the time of detection based on HbO data.

Although we just showed that the NVC response is present in the resting state and that activation in GCaMP is generally followed by an increase in HbO, this does not automatically mean that an increase in HbO is always preceded by a GCaMP spike. For this reason, we used the same spontaneous activity detection method on HbO data [Fig. 4, note: the data in A are the same as those in Fig. 2(b), with the axes altered to facilitate a direct comparison to HbO-detected activation].

Comparing the average curves around the detected spontaneous HbO increase, a larger hemodynamic but smaller GCaMP response can be seen. Furthermore, the oscillating effect of HbO has disappeared in favor of an increase of HbO before the GCaMP curve shows a clear divergence from zero. On average, over the whole brain, a detected spontaneous HbO activation was preceded by a GCaMP spike that went over the detection threshold in 50.5% of the cases (Sham: 49.6%, CaCl: 51.5%). On the other hand, detected spontaneous GCaMP activations were followed by a detected HbO activation 10.8% of the time (Sham: 10.6%, CaCl: 11.1%) [Figs. 8(c) and 8(d)]. However, this can be partially explained by the difference in the number of detected spontaneous activations; GCaMP crossed the threshold ∼10 times more often than HbO [Figs. 8(a) and 8(b) in Appendix B]. To make a fair comparison, the number of “matches” was also calculated on random activation data. This means that for the GCaMP-detected data, the same number of activations per pixel was taken, but the timing of it was determined randomly instead of being based on the data crossing the activation threshold. The number of detected activations of the HbO data was then calculated on this randomly timed data. A similar calculation was made on the HbO data, with preceding GCaMP activation detection. For these random cases, random GCaMP timepoints were followed by HbO activations 5.1 % of the time (Sham: 5.2%, CaCl: 5.0%), whereas random HbO timepoints were preceded by GCaMP activations 25.8% [Sham: 25.7%, CaCl: 26.0%, Figs. 8(e) and 8(f) in Appendix B].

### 3.3 Neurovascular Coupling with Rigidified Artery

**Fig. 5:**
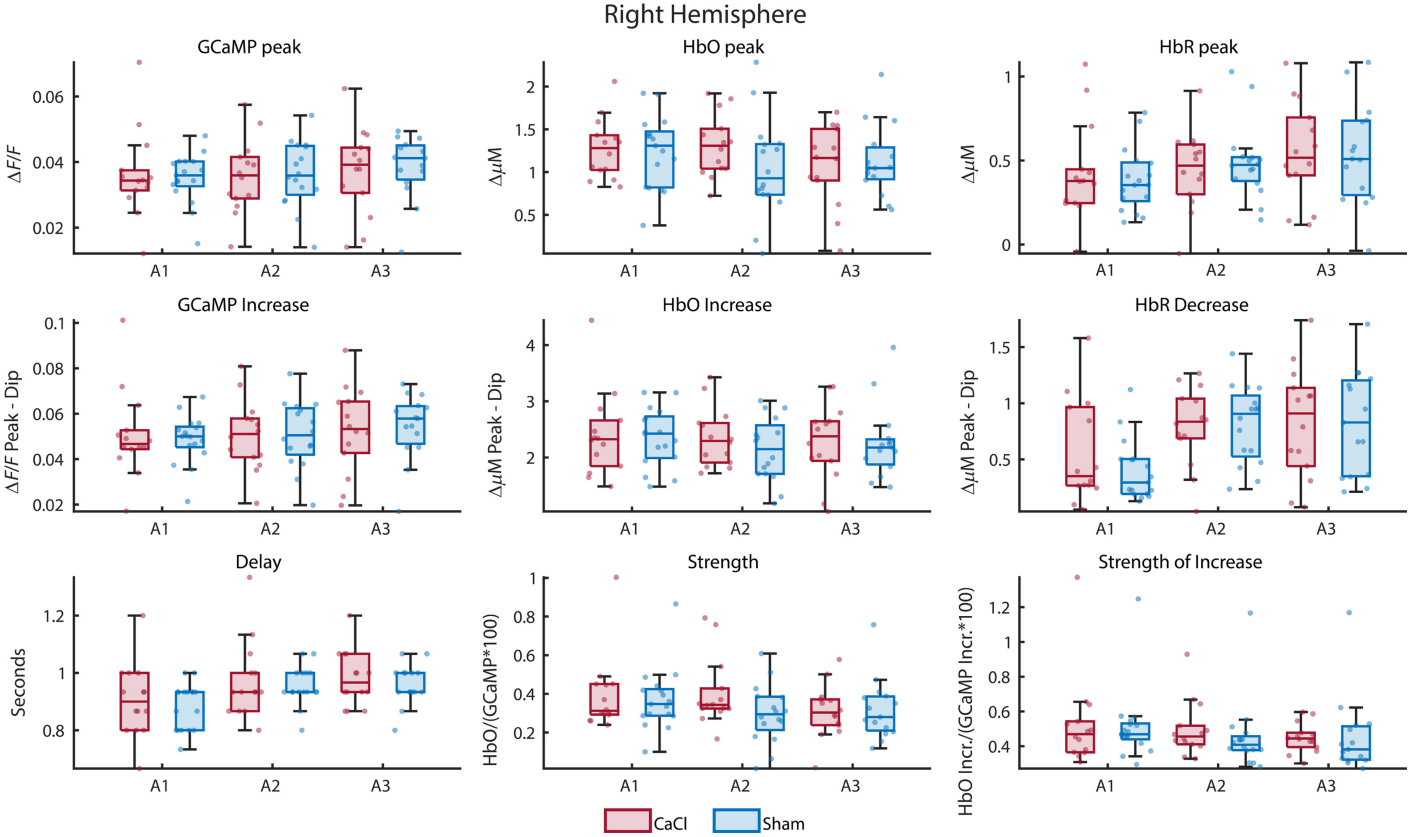
Parameters related to NVC in CaCl versus Sham-treated mice over three acquisitions. Values were taken from the right hemisphere. The same parameters as in Fig. 3 were calculated. None of the parameters showed a significant difference between the groups (Table 2 in Appendix G). For differences per brain area, see Fig. 11 in Appendix F.

Ultrasound measurements on the diameter change of the right carotid artery were done on a subset of the mice and showed significantly less diameter change during the heart pressure pulse for the CaCl group (Fig. 9 in Appendix C, p=0.04). As with the calculations on NVC in Sham, the spontaneous activations of GCaMP were detected, and the timecourses of GCaMP, HbO, and HbR from 5 s before to 10 s after the events were averaged over a region of interest. As the calcification surgery was performed on the right carotid, our region of interest is the right hemisphere (Fig. 5; for other brain regions, see Fig. 11 in Appendix F), and the possible differences between the CaCl and Sham groups were investigated. The same parameters as shown in Fig. 3 were calculated. Statistics show no significant difference between CaCl and Sham groups, and thus, no post-hoc analysis was performed (Table 2 in Appendix G).

### 3.4 Resting State Networks

**Fig. 6:**
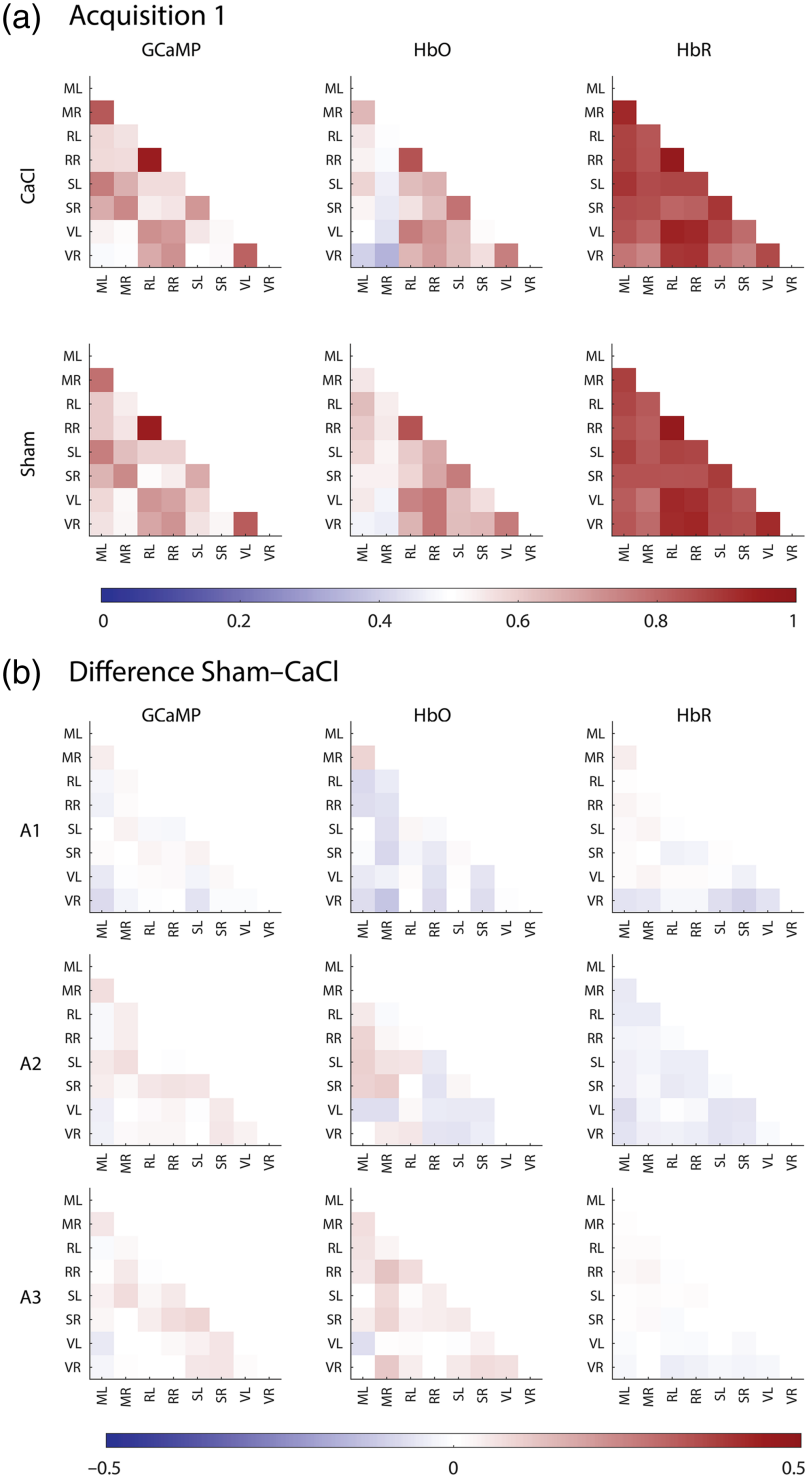
(a) Average correlation scores between seeds in different brain areas of the CaCl group (top row) and Sham group (bottom row) for GCaMP (left), HbO (middle), and HbR (right) during acquisition 1 (other acquisitions can be found in Figs. 12 in Appendix H and Fig. 13 in Appendix I). (b) The differences between the average correlation scores of the Sham group minus the CaCl group over the three acquisitions (rows). None of the datatypes (GCaMP, HbO, and HbR) nor acquisition times showed a significant difference between the Sham and CaCl groups (Tables 3 and 4 in Appendix J).

To assess the general connectivity of the brain, the timecourses of the signals localized in centroids from the regions of interest [Fig. 1(f)] were correlated with each other to produce correlation matrices for GCaMP, HbO, and HbR [Fig. 6(a) and Figs. 12 and 13 in Appendices H and I]. We observed a similar pattern in the correlation of the GCaMP and hemodynamic data. Homotopic seeds were strongly correlated, with the retrosplenial seeds in particular showing a strong correlation in all modalities, which are not only homotopic but also very close in proximity, and thus relatively “cost-effective” in their wiring.^53^ Retrosplenial and visual areas also tend to be strongly correlated to each other, whereas weaker connectivity was found between visual and motor areas. Differences between modalities can also be found. The general connectivity between seeds derived from HbR is much higher than that in GCaMP or HbO. Interestingly, where sensory and motor seeds measured with GCaMP showed a strong correlation, this correlation was a lot weaker in the hemodynamic components. Last, the correlation matrices were very robust over time, with no significant difference over the three acquisitions (Table 4 in Appendix K).

None of the seedpair connections were greatly altered in the CaCl group versus the Sham group [Fig. 6(b)]. Statistics show no significant differences in any of the ROI pairs between the two groups in any of the three acquisition times and modalities (Tables 3 and 4 in Appendix J). Even before multiple testing correction (FDR), only two seed pairs of GCaMP data showed a p-value below 0.05 in acquisition 3 (RR-SR: p=0.049 and SL-SR: p=0.028). This significance disappeared after FDR (RR-SR: q=0.554 and SL-SR: q=0.554), and no significance was found in other modalities or acquisition times.

## 4 Discussion

### 4.1 NVC in Resting State in Sham

#### 4.1.1 General NVC Observations

Indications of neurovascular coupling in resting state have been found in previous studies^10^^,^^12^^,^^41^^,^^54^^,^^55^ as well as in the current one. Ma et al.^12^ showed a clear neurovascular coupling in the resting state, and our results coincide very well with theirs, in both curve-shape and timing. In addition, Bruyns-Haylett et al.^10^ found hemodynamic curves as a response toward detected spontaneous activations that resemble our results very closely, albeit with a slower response. This difference might be explained by the presence of anesthesia in their study, which can alter the response.^52^^,^^56^ We found a delay of roughly 1 s between the HbO and GCaMP peaks, similar to other resting state studies and ultrashort stimuli findings.^12^^,^^57^^,^^58^ Studies with stimulations often show a later peak in HbO.^59^^–^^61^ This is likely due to the stronger or prolonged neuronal response that is evoked in comparison to the resting state, requiring a greater influx of HbO that in turn takes longer to reach.

In the GCaMP-detected spontaneous activation data, we can see oscillations in both GCaMP and hemodynamic data. In general, GCaMP activation fluctuates around a baseline. Because of this, a spontaneous activation in a resting state recording is likely preceded by a dip in activity, and vice versa. Although a stimulus can cut into the timecourse and “demand” an activation at any point, the spontaneous activation will rely on the fluctuations around the baseline and build upon the natural increase to reach the threshold. When the spontaneous fluctuation frequency of neurons differs by even a small amount, eventually, the timecourses will fall out of sync and cancel each other out, which will result in an average that is back at baseline.

This oscillation is seen not only in GCaMP but also in hemodynamic data. The dip before the spontaneous neuronal activity is occasionally seen in other works. In our current results, the initial dip is clear, and the results resemble the preliminary results of Bruyns–Haylett very closely.^10^ We can clearly distinguish it in Ma et al.^12^ as well, although to a lesser extent. In Wright et al.,^62^ we can spot it in the example of a single timecourse. On the other hand, the undershoot after the HbO influx is much more often observed.^10^^,^^12^^,^^54^^–^^58^^,^^61^ This dip only seems to occur in detected spontaneous activations^10^^,^^12^^,^^52^^,^^55^ and very short and/or weak stimuli^52^^,^^54^^–^^58^^,^^61^ and disappears if the stimuli get stronger.^10^^,^^56^^,^^61^

In the HbO-detected spontaneous activations, the HbO increases several seconds before crossing the threshold. This might be due to slow-wave, background fluctuations of HbO, that have a slower frequency than the response curve toward GCaMP. The threshold of HbO detection might only be reached if both the slow wave and higher-frequency fluctuations are at their peak.

#### 4.1.2 Left versus right brain area differences

When comparing the strength of the GCaMP activations above the detection threshold, we found significantly different responses between brain areas between all homotopic areas, with the right area showing significantly higher peaks than the left. A possible explanation for this could be the imaging setup. Mice were always positioned in the same manner. The location of the researcher, the angle of the LEDs to activate the GCaMP and measure the hemodynamics (acquisitions were otherwise performed in a dark room), and other factors could have had an effect on the acquisition. Interestingly, the number of times that GCaMP timecourses cross the threshold in the different areas does not show this pattern with the same intensity (Fig. 8 in Appendix B).

Although the GCaMP peaks are generally higher on the right side of the brain, the hemodynamics do not show this same pattern, with the homotopic regions differing significantly in neither HbO/HbR peak, nor increase/decrease between left and right regions. With a difference in GCaMP activity, but none in hemodynamics, we logically end up with a different strength in neurovascular coupling between right and left areas. This is more visible when looking at the strength as calculated with the increases, with all areas except for the retrosplenial showing a significantly stronger NVC in the left areas compared with the right.

#### 4.1.3 Medial versus lateral brain area differences

As regards the timing differences between brain areas, we found the retrosplenial cortex to have a significantly slower response than most other brain areas. The retrosplenial areas also have the highest HbR peak and the biggest HbR decrease. Last, we can generally distinguish two groups in the HbO data: the motor and retrosplenial areas with a lesser response, and the sensory and visual areas with a greater response, although these differences are not always significant. The difference in timing, HbO influx, and HbR peak might all stem from the same phenomenon. The vascular density is not homogeneous over the brain.^63^ The cerebral arteries feeding large parts of the more superficial parts of the brain wrap around the brain and come in laterally. This means that the middle cerebral artery comes across the sensory region, and the posterior cerebral artery feeds the visual area. The motor region and the retrosplenial area are located more medially, which might mean that the blood supply to those areas is less efficient, and thus, the delay is longer, and the peak in HbO values is lower. This would also explain the higher HbR peak; as we can see in the delay, the sensory and visual areas receive the needed HbO increase significantly quicker than the retrosplenial areas, whereas the motor areas stay in between those values.

### 4.2 Resting State Networks in Sham

RSN studies have gained interest over the years. First, RSN information is especially useful in cases where the subject cannot be asked to perform a task, for example, coma patients or young infants. Second, RSN has been found to be disrupted in many diseases affecting brain functions such as Alzheimer’s,^39^^,^^64^^,^^65^ schizophrenia,^66^ obstructive sleep apnea,^67^ and autism,^68^ among others. This opens the possibility for the usage of RSN as a biomarker to distinguish between mild cognitive impairment and Alzheimer’s,^37^^,^^38^ or even a predictive factor for Alzheimer’s.^39^

Most of these findings have been obtained with fMRI and are generally assumed to reflect ongoing neuronal processes. Our results show that this assumption rings true in general but with some subtle differences between the two networks that should not be ignored. The clearest difference lies in the connection between sensory and motor areas, which is much higher in GCaMP compared with HbO. This could be related to the difference in neurovascular coupling between these two areas. In general, the motor areas show a lower GCaMP peak, lower HbO influx, larger HbR peak, and slightly (not significantly) slower response than the sensory areas.

To prevent the accidental removal of relevant signals, a global signal regression (GSR) was not performed on the data in this article. However, as global factors such as changes in LED intensity can confound the data, correlation matrices after GSR are shown in Fig. 13 in Appendix I. The results for both methods come to the same conclusion.

### 4.3 Calcium Rigidification Effect

In this study, we used the mouse model as proposed by Sadekova et al.^40^ They demonstrated the efficiency of their method by showing an increase in carotid intima-media thickness, an increase in stiffness of the artery with a pressure myograph, and an increase in minimum to maximum speed of blood flow with a doppler OCT. Furthermore, they showed that the carotid artery radius was not different, and there was no detectable increase in systolic blood pressure compared with Sham mice. They performed these tests two weeks after the surgery. In the current study, ultrasound imaging on the carotid artery was done 3 weeks after the surgery on a subset of mice to validate that the rigidification surgery had the desired effect, which showed a significant difference between the two groups 3 weeks after the surgery. Although a limitation of this study is a lack of histological sampling and invasive verification techniques to validate the calcification surgery, in our hands, the previous success rate for the surgery was found in excess of 95% suggesting that, if any, procedural variability should be small. Previous studies with this mouse model showed a decreased resting cerebral blood flow (CBF) in perirhinal and entorhinal cortices, parts of the hippocampus (CA1 and dentate gyrus) and the thalamus,^29^ a decrease in cerebral vessel density in the somatosensory cortex and CA1 but not in the dentate gyrus,^29^ and cerebral gliosis in the hippocampus.^69^ Though the impact on the hippocampus seems clear, the current study focused on the effects on cortical regions. Given the impact on at least some cortical structures, as mentioned above, we hypothesized that the calcification of the carotid artery would have a detectable effect on the neurovascular coupling and resting state connectivity of cortical regions. Acquisitions were taken at three timepoints to track the effects of the surgery over time as the potential NVC dysfunction as a result of a rigidified artery might only appear once the microvasculature is sufficiently damaged. However, the neurovascular coupling showed no significant difference in any of the measured parameters as an effect of rigidification of the carotid artery. Similarly, none of the seedpair connections or the resting state network were significantly altered in the CaCl group versus the Sham group, indicating that a (subtle) increase in carotid artery rigidity does not alter the resting state connectivity of cortical brain areas.

## 5 Conclusion

Our research indicates that hemodynamic signal remains a solid indicator of neuronal activation in the resting state in mild cases of rigidification of the carotid artery as both resting state networks and neurovascular coupling parameters indicate no differences between Sham and CaCl groups. Moreover, detected peaks in hemodynamic data are frequently preceded by detected peaks in GCaMP data in both CaCl and Sham mice. This confirms the validity of past and future studies relying on hemodynamic data to infer neuronal activation. However, further research is needed to verify whether more severe cases of large artery rigidification alter NVC. Last, we found subtle but significant differences between brain regions regarding resting state activations and neurovascular coupling, which can be used in future research to finetune the conclusions drawn from hemodynamic data.

## 6 Appendix A: Movement—Plot

**Fig. 7:**
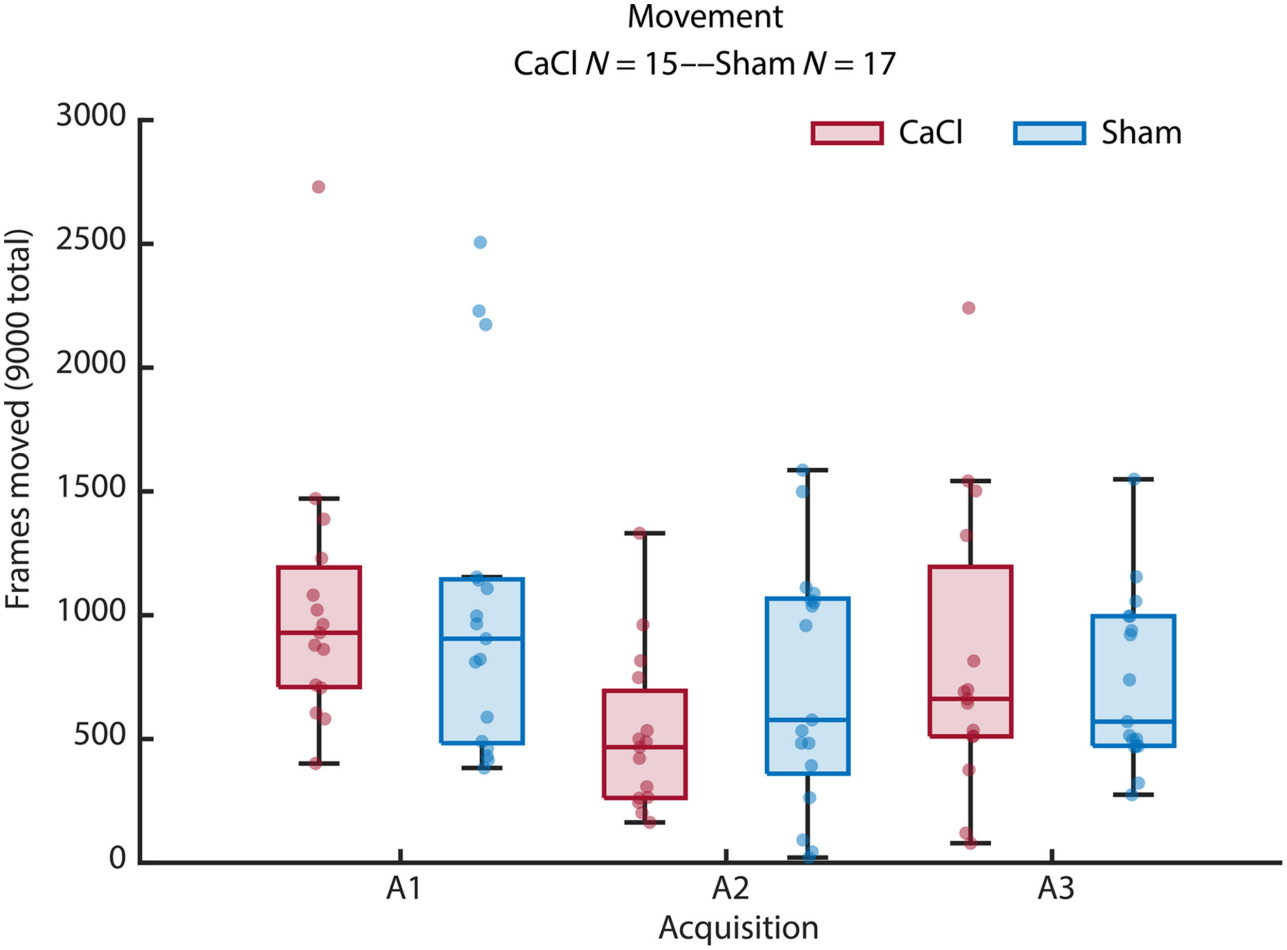
Number of frames where the mouse was moving on the treadmill. Acquisitions existed of 9000 frames in total. Movement did not differ significantly between groups in the GCaMP acquisitions (A1: p=0.37, A2: p=0.39, A3: p=0.32), as tested with Kruskal–Wallis tests.

Boxplots showing the movement of mice during imaging sessions (Fig. 7).

## 7 Appendix B: Detected GCaMP and HbO Activations

**Fig. 8:**
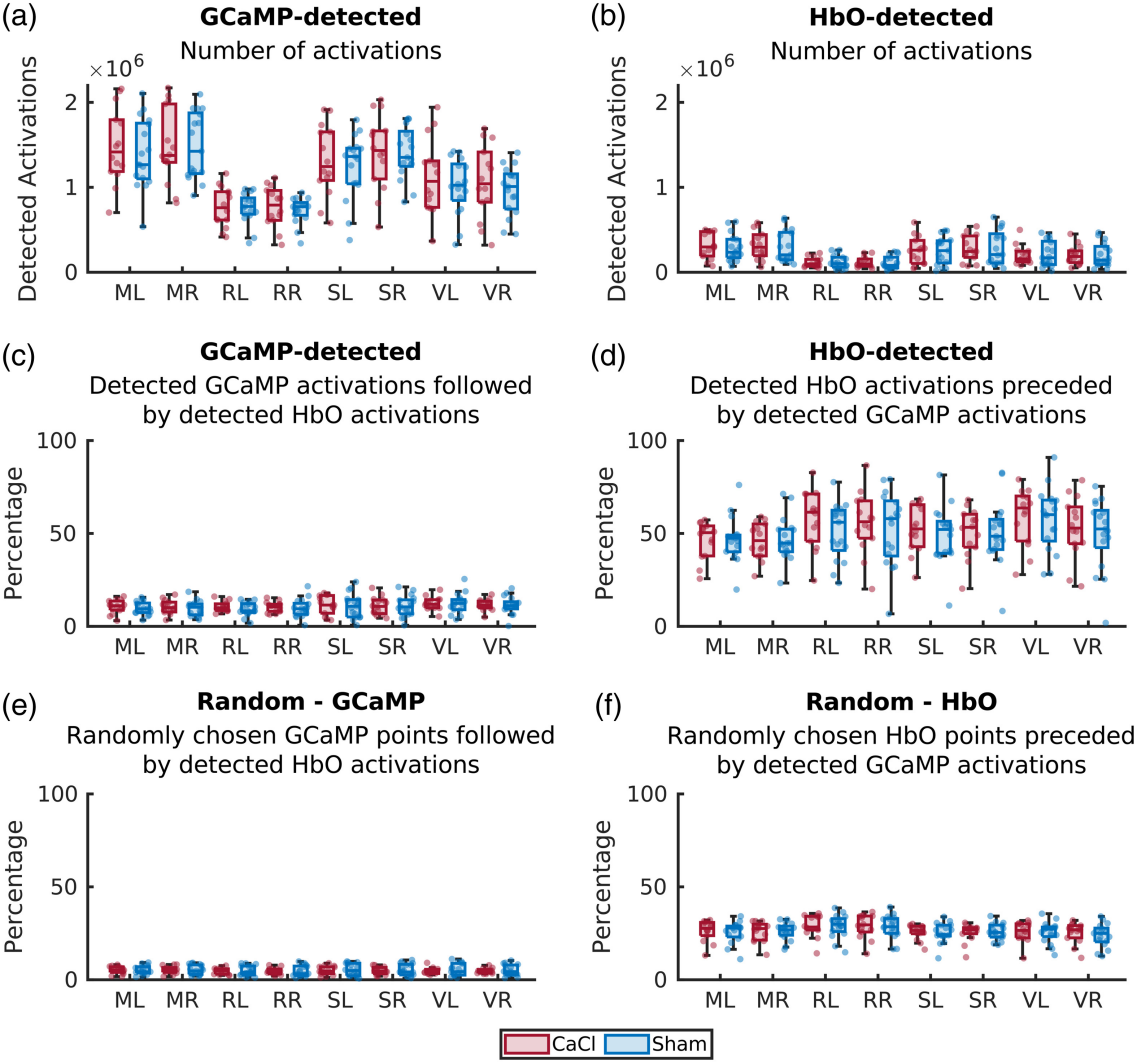
Number of spontaneous activations per brain region during acquisition 1, detected on GCaMP data (a) or HbO data (b). Per detected GCaMP activation, the percentage activations that were followed by the HbO timecourse reaching the detection threshold within 2 s are shown in panel (c). Conversely, panel (d) shows the percentage of detected HbO activations that were preceded (within 2 s) by a GCaMP peak that crossed the detection threshold. (e) The number of detected GCaMP activations of panel (a) was taken, but timepoints were randomized to fabricate a control group for panel (c). (f) Similar situation to panel (e), where the number of HbO-detected activations from panel (b) were randomized and checked for a corresponding GCaMP activation.

Spontaneous activations of GCaMP and HbO and their relationship to the other modality (Fig. 8).

## 8 Appendix C: Verification Rigidification Surgery

**Fig. 9:**
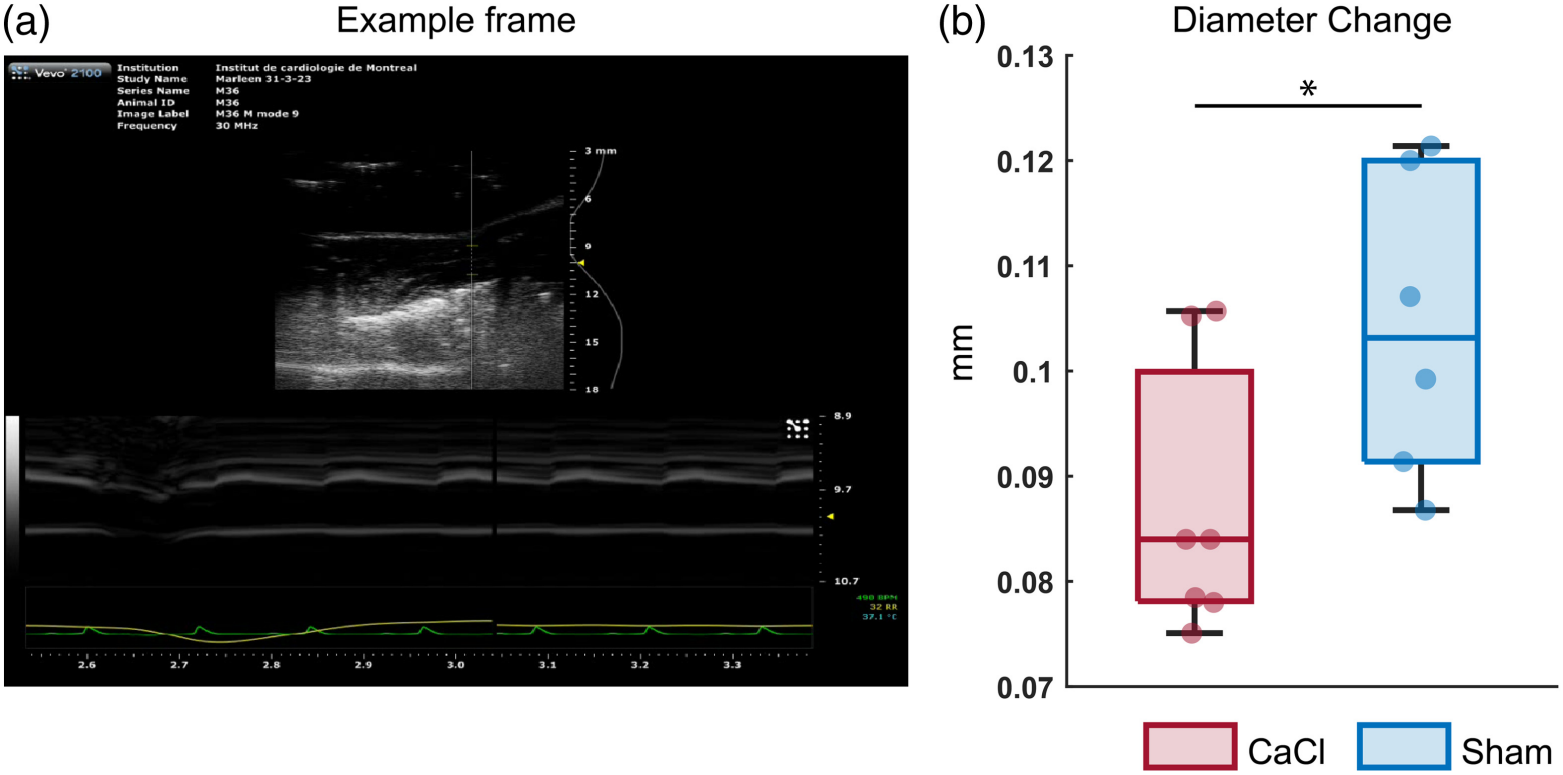
(a) Representative frame of an ultrasound recording in m-mode. (b) Diameter change of the right carotid artery. Dots indicate individual samples. N=7 for the CaCl group, and N=6 for the sham group. p=0.04.

Ultrasound results of the carotid artery of the two groups of mice (Fig. 9).

## 9 Appendix D: NVC in RS Sham—Statistics

Statistical tests regarding NVC parameters of spontaneous GCaMP activations. Tests were first conducted over all brain regions, before conducting tests per area pair to further elucidate where the differences resided (Table 1).

**Table 1:** Statistics on neurovascular coupling parameters during resting state, in Sham mice on the first acquisition. Italics indicate values <0.05. Q-values are FDR-corrected p-values. (A) Tests that were performed per parameter to see if there was a significant difference between ROIs. All parameters showed significant. (B) Post-hoc test results, showing which ROIs differed significantly per parameter.

A.										
Parameter			Testtype				p		q	
GCaMP peak			rANOVA—Greenhouse–Geisser corrected				*5.02E−12*		*2.17E−11*	
HbO peak			rANOVA—Greenhouse–Geisser corrected				*1.52E−09*		*2.28E−09*	
HbR peak			Friedman				*2.27E−12*		*2.04E−11*	
GCaMP increase			rANOVA—Greenhouse–Geisser corrected				*7.23E−12*		*2.17E−11*	
HbO increase			Friedman				*3.66E−11*		*8.22E−11*	
HbR decrease			rANOVA				*1.94E−06*		*1.94E−06*	
Delay (s)			Friedman				*2.93E−09*		*3.76E−09*	
Response strength			Friedman				*4.85E−08*		*5.45E−08*	
Response strength increase			Friedman				*7.22E−10*		*1.30E−09*	
**B.**										
**ROI 1**	**ROI 2**	**GCaMP Peak**	**HbO Peak**	**HbR Peak**	**GCaMP Increase**	**HbO Increase**	**HbR Decrease**	**Delay (sec.)**	**Response Strength**	**Response Strength Increase**
ML	MR	*4.65E−04*	1.00E+00	1.00E+00	*9.71E−04*	1.00E+00	1.00E+00	9.99E−01	6.06E−01	*4.31E−02*
ML	RL	*2.83E−04*	9.99E−01	7.44E−01	2.28E−01	9.35E−01	9.95E−01	7.63E−02	5.09E−01	1.00E+00
ML	RR	*4.24E−04*	9.38E−01	1.59E−01	*3.13E−02*	1.87E−01	6.54E−01	5.47E−02	6.48E−02	9.95E−01
ML	SL	*2.88E−02*	8.87E−01	*3.49E−02*	*1.66E−02*	*2.00E−04*	2.17E−01	9.64E−01	1.00E+00	2.88E−01
ML	SR	*5.96E−08*	8.68E−01	*1.77E−02*	*5.96E−08*	*1.44E−04*	*3.49E−02*	8.48E−01	*2.24E−02*	1.87E−01
ML	VL	2.70E−01	*1.96E−02*	5.09E−01	1.98E−01	*2.76E−04*	7.00E−01	9.98E−01	2.88E−01	1.87E−01
ML	VR	*1.47E−04*	2.92E−01	2.88E−01	1.49E−03	*1.84E−05*	9.35E−01	2.57E−01	9.67E−01	9.77E−01
MR	RL	6.24E−01	9.50E−01	8.23E−01	9.31E−01	9.35E−01	9.97E−01	2.99E−01	1.00E+00	*4.31E−02*
MR	RR	*1.94E−02*	6.96E−01	2.17E−01	7.00E−01	1.87E−01	7.00E−01	2.37E−01	9.53E−01	2.88E−01
MR	SL	9.99E−01	7.77E−01	*2.24E−02*	9.81E−01	*2.00E−04*	1.87E−01	7.15E−01	5.58E−01	*1.99E−06*
MR	SR	*5.66E−06*	6.28E−01	*1.10E−02*	*1.43E−05*	*1.44E−04*	*2.80E−02*	4.73E−01	8.23E−01	9.99E−01
MR	VL	1.00E+00	*1.73E−03*	4.15E−01	1.00E+00	*2.76E−04*	6.54E−01	9.12E−01	*9.44E−04*	*6.39E−07*
MR	VR	*8.36E−03*	1.78E−01	2.17E−01	*3.86E−02*	*1.84E−05*	9.13E−01	6.12E−02	9.95E−01	4.15E−01
RL	RR	*4.21E−03*	7.71E−01	9.77E−01	*2.89E−02*	8.88E−01	9.77E−01	1.00E+00	9.77E−01	9.95E−01
RL	SL	9.82E−01	9.48E−01	*5.26E−05*	5.01E−01	*2.24E−02*	*2.80E−02*	*2.02E−03*	4.61E−01	2.88E−01
RL	SR	*2.29E−04*	9.55E−01	*1.84E−05*	*9.54E−07*	*1.77E−02*	*2.25E−03*	*4.48E−04*	8.88E−01	1.87E−01
RL	VL	3.08E−01	*7.34E−05*	*8.53E−03*	9.04E−01	*2.80E−02*	2.17E−01	*9.10E−03*	*5.16E−04*	1.87E−01
RL	VR	*4.79E−03*	1.95E−01	*2.25E−03*	*1.90E−03*	*3.90E−03*	5.09E−01	*4.19E−06*	9.85E−01	9.77E−01
RR	SL	*3.62E−02*	1.00E+00	*3.03E−07*	9.46E−01	5.09E−01	*7.00E−04*	*1.24E−03*	5.30E−02	*4.31E−02*
RR	SR	5.25E−01	1.00E+00	*1.20E−07*	*2.98E−02*	4.61E−01	*2.62E−05*	*2.63E−04*	1.00E+00	6.54E−01
RR	VL	*3.37E−04*	7.20E−02	*1.44E−04*	3.41E−01	5.58E−01	*1.40E−02*	*5.90E−03*	*4.17E−06*	*2.24E−02*
RR	VR	2.81E−01	3.04E−01	*2.62E−05*	*2.73E−02*	2.17E−01	6.48E−02	*2.19E−06*	5.58E−01	1.00E+00
SL	SR	*3.24E−05*	1.00E+00	1.00E+00	*1.16E−04*	1.00E+00	9.97E−01	1.00E+00	*1.77E−02*	*3.72E−05*
SL	VL	9.74E−01	6.54E−01	9.35E−01	9.68E−01	1.00E+00	9.95E−01	1.00E+00	3.27E−01	1.00E+00
SL	VR	*2.38E−02*	8.84E−01	9.91E−01	1.08E−01	1.00E+00	9.13E−01	8.98E−01	9.53E−01	*2.24E−02*
SR	VL	*7.30E−05*	4.43E−01	8.58E−01	*1.90E−04*	1.00E+00	8.23E−01	9.95E−01	*5.85E−07*	*1.28E−05*
SR	VR	1.00E+00	7.99E−01	9.67E−01	1.00E+00	1.00E+00	5.09E−01	9.81E−01	3.27E−01	7.86E−01
VL	VR	*1.54E−04*	1.00E+00	1.00E+00	*2.21E−03*	9.99E−01	1.00E+00	6.89E−01	*1.77E−02*	*1.10E−02*

## 10 Appendix E: NVC in RS Sham—Plots

**Fig. 10:**
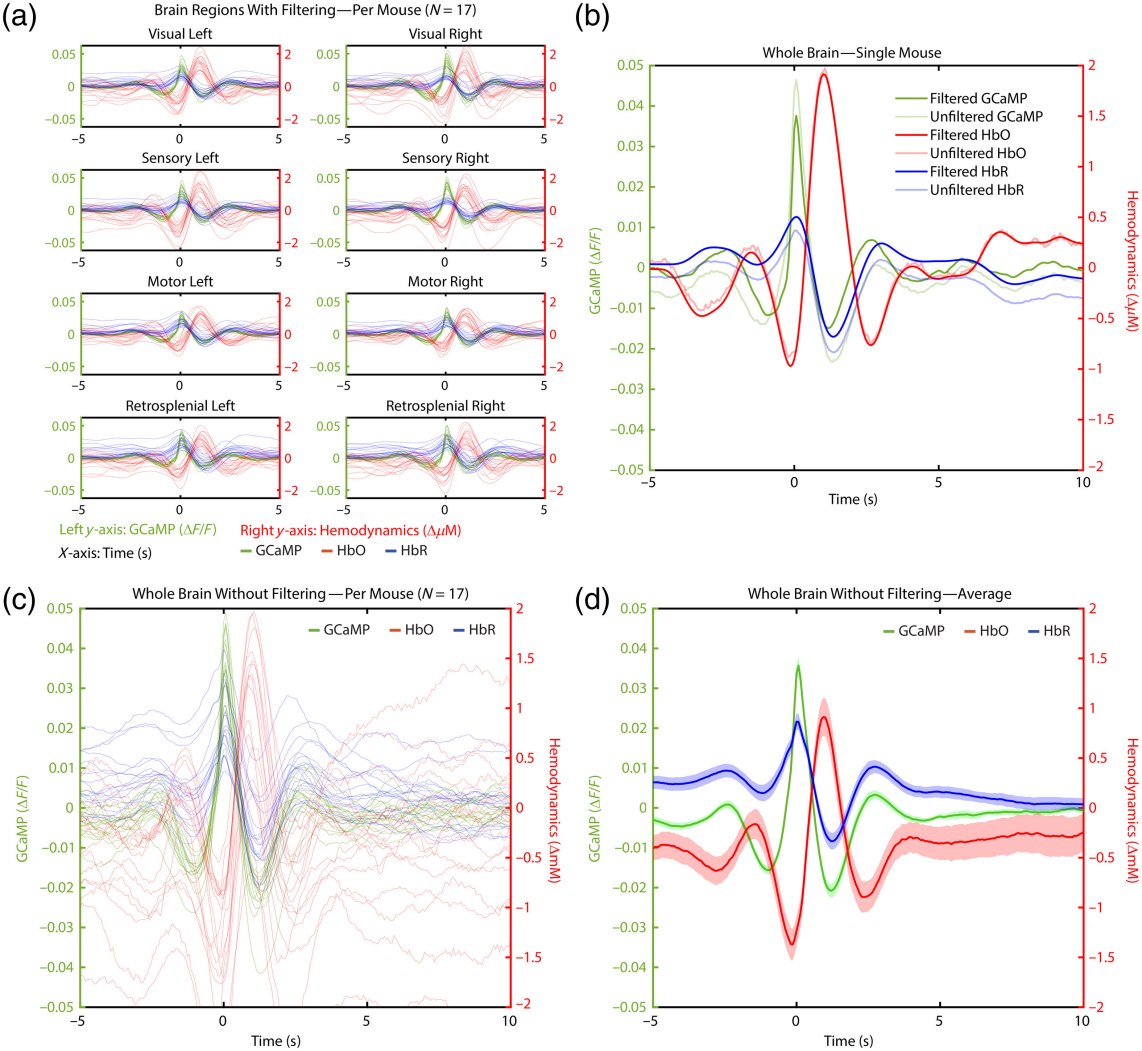
GCaMP (depicted in green) corresponds to the left y-axis, whereas hemodynamics (HbO in red and HbR in blue) correspond to the right y-axis. The x-axis shows time in seconds, with 0 being the time of the detected spontaneous GCaMP activation. (a) Curves of GCaMP and hemodynamics per mouse, per region of interest. For clarity, HbT curves are not depicted. Each line shows the average curve over a region of interest of one mouse. (b) Curves of GCaMP and hemodynamics of one mouse, over the whole brain, with filtered and unfiltered data. (c) Neurovascular coupling curves per mouse. Unfiltered version of Fig. 2(a). (d) Group average of mice depicted in panel (c). The dark line shows the group average, and the patches show the standard error of the mean (SEM). Unfiltered version of Fig. 2(b).

Unfiltered GCaMP and hemodynamic data, to illustrate that the dynamics in the filtered data are not a result of the filtering itself (Fig. 10).

## 11 Appendix F: NVC CaCl Versus Sham ROI—Figures

**Fig. 11:**
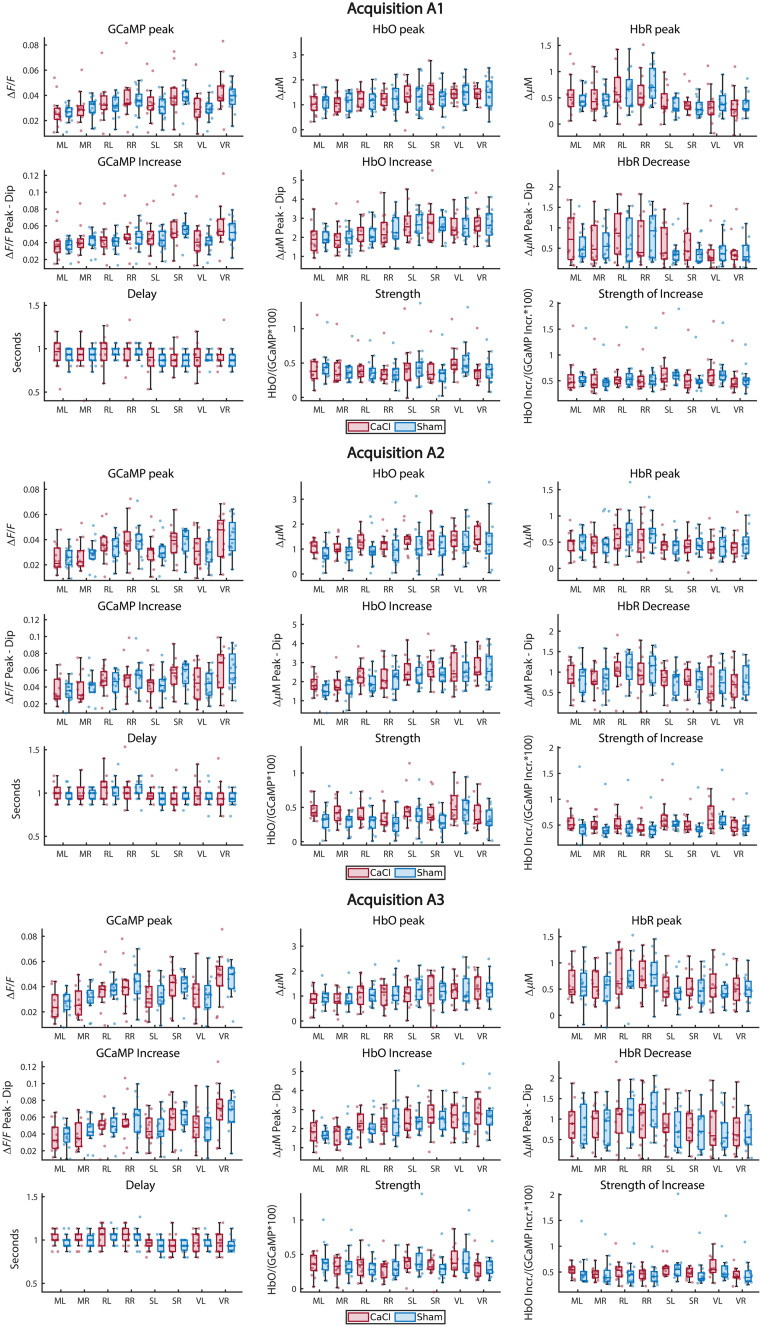
Neurovascular coupling parameters for all brain areas over the three acquisitions. For statistics on the difference between CaCl and Sham groups over the right hemisphere, see Table 2 in Appendix G.

Boxplots showing NVC parameters of all brain areas for both Sham and CaCl mice (Fig. 11).

## 12 Appendix G: NVC in CaCl Versus Sham Right Hemisphere—Statistics

Statistical tests of Sham and CaCl groups regarding NVC parameters (Table 2).

**Table 2:** Statistics for the neurovascular coupling parameters of the Sham versus CaCl groups. Q-values are FDR-corrected p-values. No significant differences were found, and thus, no post-hoc testing was done.

Parameter	Testtype	p	q
GCaMP peak	rANOVA	9.44E−01	9.79E−01
HbO peak	rANOVA	4.91E−01	9.79E−01
HbR peak	MackSkillings	3.40E−01	9.79E−01
GCaMP increase	rANOVA	9.79E−01	9.79E−01
HbO increase	rANOVA	7.15E−01	9.79E−01
HbR decrease	MackSkillings	5.43E−01	9.79E−01
Delay (s)	MackSkillings	4.34E−01	9.79E−01
Response strength	MackSkillings	8.77E−01	9.79E−01
Response strength increase	MackSkillings	7.63E−01	9.79E−01

## 13 Appendix H: Correlation Matrices Without GSR

**Fig. 12:**
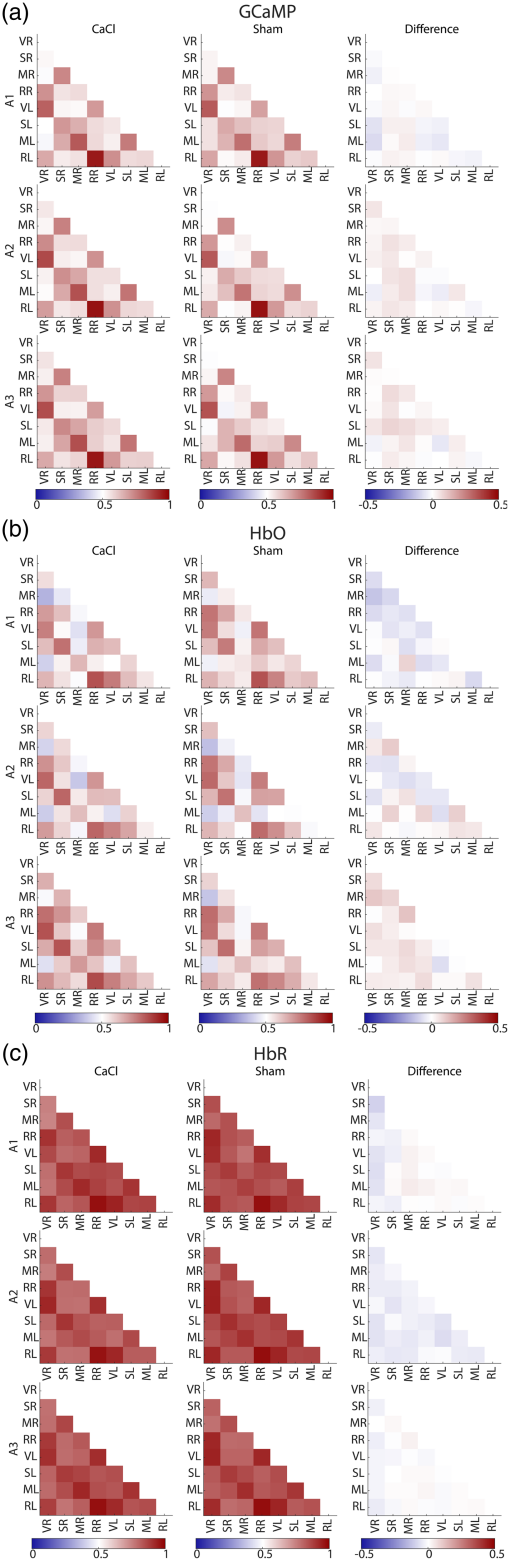
Correlation matrices of GCaMP (a), HbO (b), and HbR (c). Rows depict different acquisitions (A1, A2, and A3 for weeks 3, 4, and 5 after surgery, respectively). The first column shows the average correlation between seeds of the CaCl group, the second column shows that of the Sham group, and the third column shows the difference between Sham and CaCl (CaCl minus Sham average correlation). Differences were not significant (see Table 3 in Appendix J for statistics).

Correlation matrices over all three acquisitions and all three modalities, without GSR (Fig. 12).

## 14 Appendix I: Correlation Matrices with GSR

**Fig. 13:**
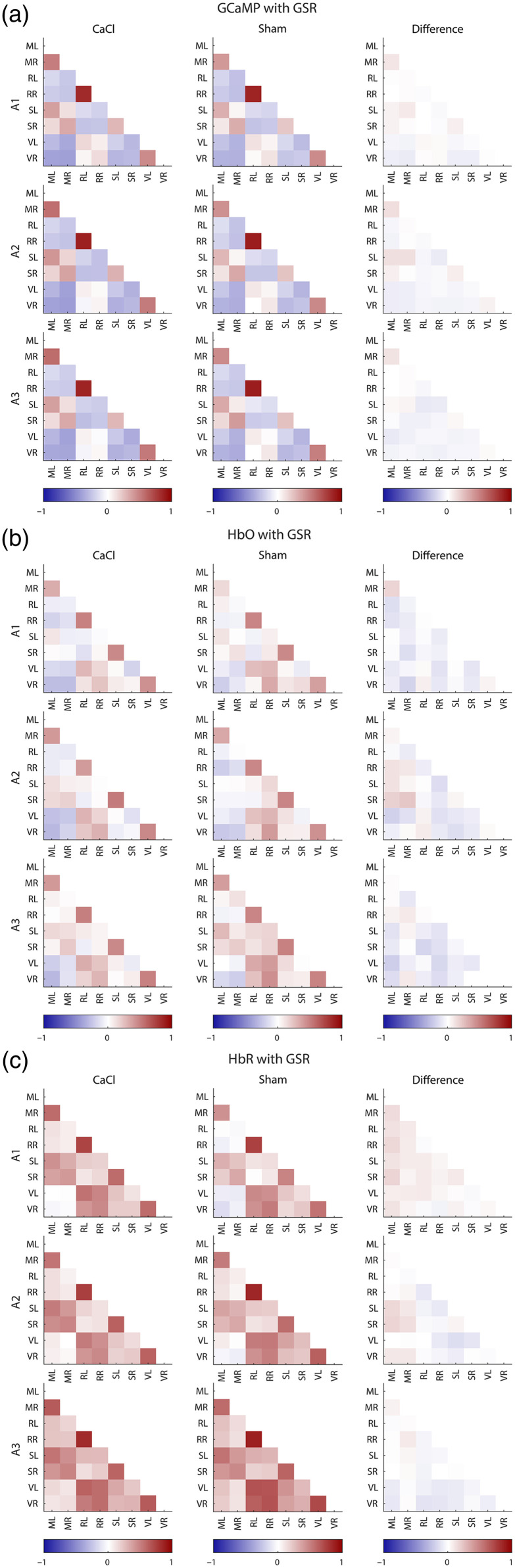
Correlation matrices of GCaMP (a), HbO (b), and HbR (c) with GSR. Rows depict different acquisitions (A1, A2, and A3 for weeks 3, 4, and 5 after surgery, respectively). The first column shows the average correlation between seeds of the CaCl group, the second column shows that of the Sham group, and the third column shows the difference between Sham and CaCl (CaCl minus Sham average correlation).

Correlation matrices over all three acquisitions and all three modalities, with GSR (Fig. 13).

## 15 Appendix J: Correlation Matrices—CaCl Versus Sham Statistics

Statistics on the differences in correlation matrices between CaCl and Sham (Table 3).

**Table 3:** Statistics of the difference between correlation matrices of CaCl and Sham groups, of GCaMP (A), HbO (B), and HbR (C), as shown in Fig. 12 in Appendix H (right columns). No GSR was performed on these data. p-Values were calculated with Mann–Whitney U tests. Bold-italics indicate values <0.05. The upper triangle (italic text) shows p-values, and the lower triangle (bold text) shows the FDR-corrected p-values; q-values.

A.									
**GCaMP**		ML	MR	RL	RR	SL	SR	VL	VR
**A1**	**ML**		*0.258*	*0.706*	*0.603*	*0.620*	*0.706*	*0.439*	*0.147*
	**MR**	**0.918**		*0.592*	*0.787*	*0.416*	*1.000*	*0.890*	*0.487*
	**RL**	**0.918**	**0.918**		*0.755*	*0.620*	*0.620*	*0.736*	*0.565*
	**RR**	**0.918**	**0.918**	**0.918**		*0.633*	*0.633*	*0.418*	*0.884*
	**SL**	**0.918**	**0.918**	**0.918**	**0.918**		*0.311*	*0.620*	*0.226*
	**SR**	**0.918**	**1.000**	**0.918**	**0.918**	**0.918**		*0.736*	*0.677*
	**VL**	**0.918**	**0.955**	**0.918**	**0.918**	**0.918**	**0.918**		*0.921*
	**VR**	**0.918**	**0.918**	**0.918**	**0.955**	**0.918**	**0.918**	**0.955**	
**A2**		ML	MR	RL	RR	SL	SR	VL	VR
	**ML**		*0.054*	*0.706*	*0.858*	*0.159*	*0.226*	*0.463*	*0.706*
	**MR**	**0.741**		*0.293*	*0.293*	*0.159*	*0.463*	*0.890*	*0.858*
	**RL**	**0.890**	**0.744**		*0.487*	*0.827*	*0.331*	*0.565*	*0.372*
	**RR**	**0.890**	**0.744**	**0.758**		*0.890*	*0.117*	*0.311*	*0.416*
	**SL**	**0.741**	**0.741**	**0.890**	**0.890**		*0.108*	*0.796*	*0.796*
	**SR**	**0.744**	**0.758**	**0.744**	**0.741**	**0.741**		*0.372*	*0.258*
	**VL**	**0.758**	**0.890**	**0.832**	**0.744**	**0.890**	**0.744**		*0.099*
	**VR**	**0.890**	**0.890**	**0.744**	**0.758**	**0.890**	**0.744**	**0.741**	
**A3**		ML	MR	RL	RR	SL	SR	VL	VR
	**ML**		*0.059*	*0.819*	*0.827*	*0.171*	*0.439*	*0.184*	*0.512*
	**MR**	**0.554**		*0.917*	*0.487*	*0.137*	*0.890*	*0.620*	*0.858*
	**RL**	**1.000**	**1.000**		*1.000*	*0.394*	*0.289*	*0.983*	*0.724*
	**RR**	**1.000**	**0.844**	**1.000**		*0.147*	***0.049***	*0.439*	*0.984*
	**SL**	**0.734**	**0.734**	**0.844**	**0.734**		***0.028***	*0.706*	*0.487*
	**SR**	**0.844**	**1.000**	**0.844**	**0.554**	**0.554**		*0.439*	*0.211*
	**VL**	**0.734**	**0.964**	**1.000**	**0.844**	**1.000**	**0.844**		*0.487*
	**VR**	**0.844**	**1.000**	**1.000**	**1.000**	**0.844**	**0.739**	**0.844**	
**B.**									
**HbO**		ML	MR	RL	RR	SL	SR	VL	VR
**A1**	**ML**		*0.260*	*0.064*	*0.333*	*0.647*	*0.777*	*0.323*	*0.132*
	**MR**	**0.732**		*0.481*	*0.326*	*0.431*	*0.481*	*0.305*	*0.056*
	**RL**	**0.732**	**0.748**		*0.442*	*0.767*	*0.828*	*0.340*	*0.567*
	**RR**	**0.732**	**0.732**	**0.748**		*0.724*	*0.205*	*0.285*	*0.109*
	**SL**	**0.862**	**0.748**	**0.906**	**0.906**		*0.921*	*0.934*	*0.953*
	**SR**	**0.906**	**0.748**	**0.927**	**0.732**	**0.953**		*0.619*	*0.213*
	**VL**	**0.732**	**0.732**	**0.732**	**0.732**	**0.953**	**0.862**		*0.455*
	**VR**	**0.732**	**0.732**	**0.835**	**0.732**	**0.953**	**0.732**	**0.748**	
**A2**		ML	MR	RL	RR	SL	SR	VL	VR
	**ML**		*0.348*	*0.418*	*0.220*	*0.260*	*0.169*	*0.369*	*0.859*
	**MR**	**0.688**		*0.777*	*0.945*	*0.305*	*0.148*	*0.320*	*0.983*
	**RL**	**0.688**	**0.946**		*0.662*	*0.395*	*0.879*	*0.645*	*0.109*
	**RR**	**0.688**	**0.980**	**0.905**		*0.350*	*0.183*	*0.067*	*0.329*
	**SL**	**0.688**	**0.688**	**0.688**	**0.688**		*0.678*	*0.263*	*0.374*
	**SR**	**0.688**	**0.688**	**0.946**	**0.688**	**0.905**		*0.826*	*0.879*
	**VL**	**0.688**	**0.688**	**0.905**	**0.688**	**0.688**	**0.946**		*0.469*
	**VR**	**0.946**	**0.983**	**0.688**	**0.688**	**0.688**	**0.946**	**0.730**	
**A3**		ML	MR	RL	RR	SL	SR	VL	VR
	**ML**		*0.369*	*0.182*	*0.344*	*0.939*	*0.544*	*0.320*	*0.865*
	**MR**	**0.854**		*0.678*	*0.140*	*0.421*	*0.182*	*0.818*	*0.088*
	**RL**	**0.788**	**0.863**		*0.128*	*0.678*	*0.580*	*0.782*	*0.249*
	**RR**	**0.854**	**0.788**	**0.788**		*0.643*	*0.434*	*0.311*	*0.712*
	**SL**	**0.973**	**0.854**	**0.863**	**0.863**		*0.479*	*0.981*	*0.510*
	**SR**	**0.854**	**0.788**	**0.855**	**0.854**	**0.854**		*0.549*	*0.197*
	**VL**	**0.854**	**0.916**	**0.913**	**0.854**	**0.981**	**0.854**		*0.097*
	**VR**	**0.932**	**0.788**	**0.854**	**0.867**	**0.854**	**0.788**	**0.788**	
**C.**									
**HbR**		ML	MR	RL	RR	SL	SR	VL	VR
**A1**	**ML**		*0.649*	*0.177*	*0.525*	*0.707*	*0.567*	*0.767*	*0.109*
	**MR**	**0.957**		*0.493*	*0.878*	*0.921*	*0.489*	*0.465*	*0.072*
	**RL**	**0.644**	**0.908**		*0.584*	*0.819*	*0.350*	*0.253*	*0.152*
	**RR**	**0.908**	**0.992**	**0.908**		*0.983*	*0.142*	*0.196*	*0.228*
	**SL**	**0.990**	**0.992**	**0.992**	**1.000**		*1.000*	*0.797*	*0.244*
	**SR**	**0.908**	**0.908**	**0.816**	**0.644**	**1.000**		*0.118*	*0.101*
	**VL**	**0.992**	**0.908**	**0.644**	**0.644**	**0.992**	**0.644**		*0.890*
	**VR**	**0.644**	**0.644**	**0.644**	**0.644**	**0.644**	**0.644**	**0.992**	
**A2**		ML	MR	RL	RR	SL	SR	VL	VR
	**ML**		*0.323*	*0.182*	*0.452*	*0.301*	*0.482*	*0.085*	*0.077*
	**MR**	**0.503**		*0.216*	*0.512*	*0.206*	*0.297*	*0.320*	*0.323*
	**RL**	**0.503**	**0.503**		*0.182*	*0.115*	*0.442*	*0.554*	*0.234*
	**RR**	**0.574**	**0.574**	**0.503**		*0.198*	*0.538*	*0.190*	*0.062*
	**SL**	**0.503**	**0.503**	**0.503**	**0.503**		*0.512*	*0.062*	*0.070*
	**SR**	**0.574**	**0.503**	**0.574**	**0.575**	**0.574**		*0.412*	*0.716*
	**VL**	**0.476**	**0.503**	**0.575**	**0.503**	**0.476**	**0.574**		*0.344*
	**VR**	**0.476**	**0.503**	**0.503**	**0.476**	**0.476**	**0.716**	**0.507**	
**A3**		ML	MR	RL	RR	SL	SR	VL	VR
	**ML**		*0.305*	*0.743*	*0.647*	*0.913*	*0.445*	*0.678*	*0.711*
	**MR**	**0.946**		*0.678*	*0.305*	*0.214*	*0.102*	*1.000*	*0.616*
	**RL**	**0.946**	**0.946**		*0.585*	*0.743*	*0.743*	*0.395*	*0.111*
	**RR**	**0.946**	**0.946**	**0.946**		*0.585*	*0.743*	*0.395*	*0.169*
	**SL**	**0.947**	**0.946**	**0.946**	**0.946**		*0.585*	*0.711*	*0.844*
	**SR**	**0.946**	**0.946**	**0.946**	**0.946**	**0.946**		*0.913*	*0.810*
	**VL**	**0.946**	**1.000**	**0.946**	**0.946**	**0.946**	**0.947**		*0.879*
	**VR**	**0.946**	**0.946**	**0.946**	**0.946**	**0.947**	**0.947**	**0.947**	

## 16 Appendix K: Correlation Matrices—Comparisons over Timepoints A1–A2–A3

Statistics on the differences in correlation matrices over timepoints (Table 4).

**Table 4:** Statistics of the difference between correlation matrices over timepoints (A1, A2, and A3), of GCaMP (A), HbO (B), and HbR (C). p-Values were calculated with Friedman tests. Bold-italics indicate values <0.05. The upper triangle (italic) shows p-values, and the lower triangle (bold) shows the FDR-corrected p-values; q-values. Since no q-values showed significant, no post-hoc testing was performed.

A.									
**GCaMP**		ML	MR	RL	RR	SL	SR	VL	VR
	**ML**		*0.159*	*0.356*	*0.875*	*0.798*	*0.879*	*0.405*	*0.405*
	**MR**	**0.968**		*0.531*	*0.435*	*0.542*	*0.405*	*0.798*	*0.879*
	**RL**	**0.968**	**0.968**		***0.015***	*0.875*	*0.792*	*0.905*	*0.875*
	**RR**	**0.968**	**0.968**	**0.406**		*0.905*	*0.967*	*0.435*	*0.648*
	**SL**	**0.968**	**0.968**	**0.968**	**0.968**		*0.748*	*0.968*	*0.748*
	**SR**	**0.968**	**0.968**	**0.968**	**0.968**	**0.968**		*0.657*	*0.679*
	**VL**	**0.968**	**0.968**	**0.968**	**0.968**	**0.968**	**0.968**		*0.078*
	**VR**	**0.968**	**0.968**	**0.968**	**0.968**	**0.968**	**0.968**	**0.968**	
**B.**									
**HbO**		ML	MR	RL	RR	SL	SR	VL	VR
	**ML**		*0.241*	*0.459*	***0.040***	*0.651*	***0.026***	*0.340*	*0.852*
	**MR**	**0.701**		*0.630*	***0.018***	*0.891*	*0.102*	*0.499*	*0.197*
	**RL**	**0.960**	**0.960**		*0.236*	*0.867*	*0.607*	*0.607*	*0.895*
	**RR**	**0.369**	**0.368**	**0.701**		*0.862*	*0.250*	*0.882*	*0.482*
	**SL**	**0.960**	**0.962**	**0.962**	**0.962**		*0.158*	*0.962*	*0.891*
	**SR**	**0.368**	**0.701**	**0.960**	**0.701**	**0.701**		*0.568*	*0.962*
	**VL**	**0.864**	**0.960**	**0.960**	**0.962**	**0.962**	**0.960**		*0.214*
	**VR**	**0.962**	**0.701**	**0.962**	**0.960**	**0.962**	**0.962**	**0.701**	
**C.**									
**HbR**		ML	MR	RL	RR	SL	SR	VL	VR
	**ML**		*0.088*	***0.020***	*0.054*	*0.393*	*0.519*	*0.104*	*0.540*
	**MR**	**0.266**		***0.020***	***0.025***	*0.301*	*0.519*	*0.236*	*0.446*
	**RL**	**0.114**	**0.114**		*0.862*	***0.005***	*0.104*	*0.595*	*0.595*
	**RR**	**0.214**	**0.115**	**0.862**		***0.004***	***0.008***	*0.607*	*0.417*
	**SL**	**0.679**	**0.602**	**0.071**	**0.071**		*0.639*	*0.495*	*0.482*
	**SR**	**0.679**	**0.679**	**0.266**	**0.071**	**0.688**		*0.764*	*0.080*
	**VL**	**0.266**	**0.508**	**0.679**	**0.679**	**0.679**	**0.792**		*0.228*
	**VR**	**0.679**	**0.679**	**0.679**	**0.679**	**0.679**	**0.266**	**0.508**	

## Data Availability

The MATLAB code used for this project can be found at https://github.com/marl1bakker/CaCl_P2. Data will be provided by the corresponding author upon request.
